# *cis-*regulatory analysis of the *Drosophila pdm* locus reveals a diversity of neural enhancers

**DOI:** 10.1186/s12864-015-1897-2

**Published:** 2015-09-16

**Authors:** Jermaine Ross, Alexander Kuzin, Thomas Brody, Ward F. Odenwald

**Affiliations:** The Neural Cell-Fate Determinants Section, NINDS, NIH, Bethesda, MD USA

**Keywords:** Nubbin, pdm-2, cis-regulation, Neurogenesis, Drosophila, Enhancers

## Abstract

**Background:**

One of the major challenges in developmental biology is to understand the regulatory events that generate neuronal diversity. During *Drosophila* embryonic neural lineage development, cellular temporal identity is established in part by a transcription factor (TF) regulatory network that mediates a cascade of cellular identity decisions. Two of the regulators essential to this network are the POU-domain TFs Nubbin and Pdm-2, encoded by adjacent genes collectively known as *pdm*. The focus of this study is the discovery and characterization of *cis*-regulatory DNA that governs their expression.

**Results:**

Phylogenetic footprinting analysis of a 125 kb genomic region that spans the *pdm* locus identified 116 conserved sequence clusters. To determine which of these regions function as *cis*-regulatory enhancers that regulate the dynamics of *pdm* gene expression, we tested each for *in vivo* enhancer activity during embryonic development and postembryonic neurogenesis. Our screen revealed 77 unique enhancers positioned throughout the noncoding region of the *pdm* locus. Many of these activated neural-specific gene expression during different developmental stages and many drove expression in overlapping patterns. Sequence comparisons of functionally related enhancers that activate overlapping expression patterns revealed that they share conserved elements that can be predictive of enhancer behavior. To facilitate data accessibility, the results of our analysis are catalogued in *cis*Patterns, an online database of the structure and function of these and other *Drosophila* enhancers.

**Conclusions:**

These studies reveal a diversity of modular enhancers that most likely regulate *pdm* gene expression during embryonic and adult development, highlighting a high level of temporal and spatial expression specificity. In addition, we discovered clusters of functionally related enhancers throughout the *pdm* locus. A subset of these enhancers share conserved elements including sequences that correspond to known TF DNA binding sites. Although comparative analysis of the *nubbin* and *pdm-2* encoding sequences indicate that these two genes most likely arose from a duplication event, we found only partial evidence of sequence duplication between their enhancers, suggesting that after the putative duplication their *cis*-regulatory DNA diverged at a higher rate than their coding sequences.

**Electronic supplementary material:**

The online version of this article (doi:10.1186/s12864-015-1897-2) contains supplementary material, which is available to authorized users.

## Background

During *Drosophila* neuroblast (NB) lineage development, successive NB expression of the TF genes *hunchback* (*hb*) → *Krüppel* (*Kr*) → *nubbin & pdm-2* (*pdm)* → *castor* (*cas*) is required for the birth order-dependent specification of neuronal identity [1, 2]. Recent studies indicate these genes are regulated by multiple modular enhancers located in their flanking genomic regions and/or within intronic sequences [3, 4]. For example, seven enhancers that regulate *cas* gene expression dynamics have been identified [5].

During the past two decades, functional analyses of many vertebrate and invertebrate enhancers have revealed that they are made up of multiple DNA-binding sites for different TFs, which collectively regulate enhancer activity, and that combinatorial protein-DNA and protein-protein interactions play an important role in specifying enhancer regulatory behavior [6]. Phylogenetic comparative analyses of these enhancers have revealed a high degree of conservation within their sequences [7, 8]. For example, previous studies have shown that the *hb* [4] and *cas* [5] enhancers are each made up of a cluster of sequence blocks present in all drosophilids that we refer to as a conserved sequence cluster (CSC). These and other studies have shown that many of the noncoding CSCs function as autonomous *cis*-regulatory elements that control different spatial and temporal aspects of gene expression dynamics [7–9].

**Fig. 1:**
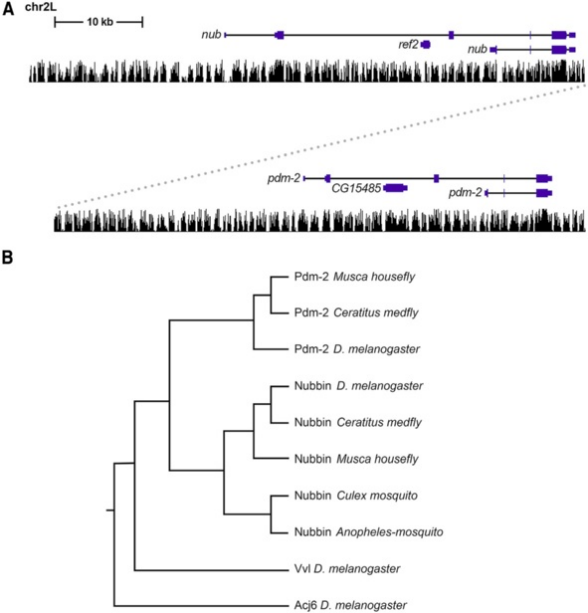
The *pdm* locus and the evolutionary relationship of its encoded *pdm* proteins. **a** An alignment of the long and short isoforms of *nub* and *pdm-2* genes to a UCSC genome browser histogram along the left arm of the 2nd chromosome (chr2L). Peaks indicate degrees of evolutionary conservation among 12 *Drosophila* species. **b** Clustal alignment of Dipteran POU protein sequences including the short isoforms of Nubbin and Pdm-2 from *D. melanogaster*, *Musca domesticus* (housefly), *Anopheles gambiae* and *Culex quinquefasciatus* (mosquito) and *Ceratitis capitata* (Mediterranean fly). The *D. melanogaster* POU homeodomain transcription factors Ventral veins lacking (Vvl) and Abnormal chemosensory jump 6 (Acj6) amino acid sequences were included as outgroup comparisons. Alignment was carried out using Clustal W2 server of Kyoto University Bioinformatics Center (http://www.genome.jp/tools/clustalw/). The tree was constructed using the UPGMA (Unweighted Pair Group Method with Arithmetic Mean) algorithm

Located on the left arm of the 2nd chromosome, the adjacent *pdm* genes encode POU homeodomain TFs that are essential for neurogenesis [10–12] (Fig. 1a). The abundance of CSCs flanking the *pdm* genes and the dynamic nature of their expression [3, 10, 12] indicate that the *pdm* locus may contain multiple enhancers that regulate different or overlapping temporal and/or spatial aspects of their expression. Previous analysis of *pdm* gene regulation has identified three enhancers that recapitulate limited *pdm* expression in a subset of cells in the cellular blastoderm [3] and within the embryonic CNS [3, 9]. Given that these enhancers activate expression in only a subset of the tissues known to express the *pdm* genes, we set out to identify *pdm* locus enhancers that may regulate other aspects of *pdm* expression.

Our phylogenetic footprint analysis of the 125 kb *pdm* locus using 12 drosophilids identified 116 CSCs (both coding and noncoding). Enhancer-reporter transgene analysis of these CSCs revealed 77 distinct *cis*-regulatory modules that activate reporter expression in different temporal and spatial subsets of the *pdm* expression domain. Although *nubbin (nub)* and *pdm-2* most likely arose from a duplication event, we found little evidence of sequence collinearity between their noncoding sequences. However, *cis*-regulatory analysis of the CSCs flanking *pdm* shows that they each contain a diversity of functionally related enhancers. Comparative analysis of these enhancers revealed the presence of multiple conserved elements within them, and many of these correspond to consensus DNA-binding motifs for different TFs, including Hb [13, 14] and Cas [3]. In addition, our analysis demonstrated clustering of functionally related enhancers, such as those that direct expression in the adult subesophageal ganglion (SOG), and we found that the functional relationship of the SOG enhancers can be inferred based on their shared conserved sequence elements. To increase the accessibility of the enhancer GAL4 transformant lines to the scientific community and enhance the description of the *pdm* locus *cis*-regulatory data, we have developed an online database that catalogues the *pdm* locus enhancers and highlights *in vivo cis*-regulatory activity in addition to their conserved sequences.

## Results

### Sequence conservation analysis within the Dipteran *pdm* locus

In *Drosophila*, the *pdm* genes share a similar exon-intron gene structure and are positioned adjacently on the left arm of the 2nd chromosome at cytological map position 33F1 [10]. Both paralogs have long and short isoforms, and each has five exons (Fig. 1a). Given the overall exon-intron organization of these genes and their homologous amino acid sequences, they most likely arose from a duplication event that occurred before *Drosophila* speciation, since all drosophilids contain both tandemly linked genes [12, 15]. The availability of genomic sequences from other Diptera, including 24 mosquito species, the Mediterranean fruit fly (*Ceratitis capitata* or medfly) and the housefly (*Musca domestica*), has allowed us to compare the Nub and Pdm-2 proteins in each of these species and determined their sequence relationship (Fig. 1b). Blastp alignment data reveals that the short isoforms of both Nub and Pdm-2 are present in both the housefly and medfly. In contrast, only a single Pdm ortholog is present in the mosquito, and comparative protein analysis indicates that its sequence aligns more closely to Nub (Fig. 1b). This indicates that either the mosquito lost one of the *pdm* genes or that the duplication occurred prior to the divergence of *Drosophila* from medfly and housefly (~100 million years ago) [16] but more recent than the divergence of *Drosophila* from mosquitos (~260 million years ago) [17]. Sequence alignments also reveal a high degree of conservation between the *Drosophila nub* and *pdm-2* 3’ exons that code for their POU domain and homeodomain. In contrast, we were unable to align the 5’ exons of either the long or short *pdm* isoforms, indicating extensive sequence divergence within the N-terminal domains of these proteins. We also found a lack of detectable DNA sequence relationship between the *nub* and *pdm-2* noncoding sequences (both conserved and less conserved sequences) using the pairwise sequence alignment tools Blastn [18] and *cis*-Decoder [8]. Taken together, our findings reveal that the collinearity between the *pdm* genes is largely restricted to their POU domain and homeodomain coding sequences, whereas the remaining portions of the *pdm* genes have undergone significant divergence from one another.

**Fig. 2:**
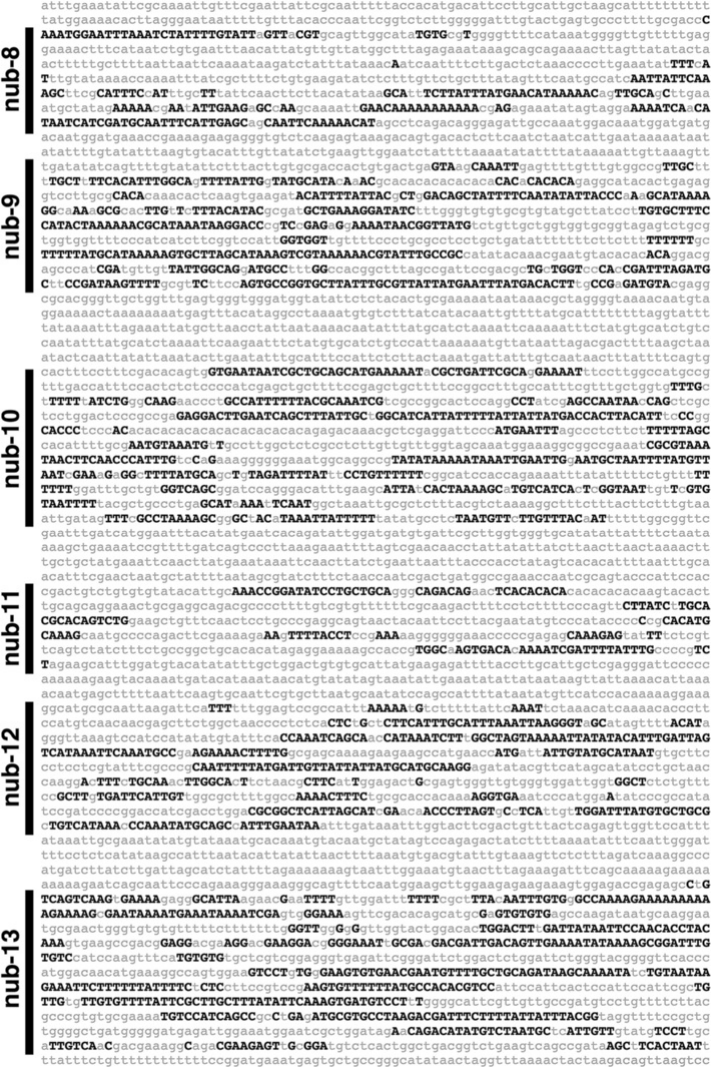
*EvoPrint* analysis of the *Drosophila pdm* locus reveals multiple noncoding sequence clusters conserved in drosophilids. Shown is a 6.2 kb region located 22.3 kb upstream to the predicted transcription start site of the *nub* long transcript that corresponds to the genomic region spanning nub-8 through nub-13 conserved sequence clusters (also illustrated in Fig. 3 and Additional file 6: Figure S4). *Black capital letters* represent *D. melanogaster* bases conserved in *D. simulans, D. sechellia, D. yakuba, D. erecta, D. ananassae, D. persimilis, D. pseudoobscura, D. willistoni, D. virilis, D. mojavensis and D. grimshaw* orthologous DNA sequences. *Lowercase gray letters* represent bases not shared by all 12 *Drosophila* species included in the analysis

**Fig. 3:**
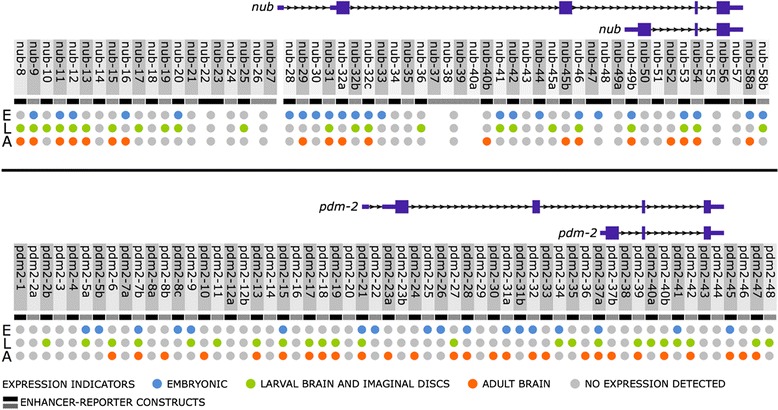
Multiple *pdm* locus enhancers regulate transgene expression in embryonic, larval, and/or adult tissues. Shown is a summary of *cis*-regulatory activity of the 116 conserved sequence clusters (CSCs) during three developmental stages. The schematic representations of the *nub* and *pdm-2* gene structures are aligned to fragments that were tested for *cis-*regulatory activity (*alternating black* and *grey blocks*, see Methods). The three developmental stages tested for enhancer activity are shown in vertical rows (embryonic, E; larval CNS, L; and adult brain, A) along with expression indicators (embryonic, *blue*; larval CNS, *green*; adult brain, *orange*; or no expression detected, *grey*). Note that nub-58b is immediately adjacent to pdm2-1. The length of the *nub* and *pdm-2* loci are not drawn to scale

As an initial step toward identifying *cis*-regulatory sequences that may control the dynamics of *pdm* gene expression, we surveyed the *D. melanogaster pdm* locus and its flanking sequences that span 125 kb positioned between a 7 kb transposable element ~29 kb upstream of the *nub* transcription start site and a chaperonin-encoding gene (*CG5525*) immediately downstream of *pdm-2*. We identified conserved sequences by phylogenetic footprinting using alignments of 12 *Drosophila* species, including *D. melanogaster*, *D. simulans, D. sechellia, D. yakuba, D. erecta, D. ananassae, D. persimilis, D. pseudoobscura, D. willistoni, D. virilis, D. mojavensis and D. grimshaw*. Our comparative analysis revealed multiple highly conserved sequence clusters that have undergone a cumulative evolutionary divergence of >150 million years [8]. The comparative analysis identified 116 CSCs (both coding and noncoding) within the *pdm* locus (Figs. 2 and 3).

As indicated above, pairwise DNA alignments of flanking and intergenic *pdm* sequences did not show any evidence of collinearity. For example, the number of CSCs within the *nub* and *pdm-2* introns differ. We identified 16 CSCs within the first intron of the *nub* long isoform transcript, and only 9 CSCs between the first and second exons of the *pdm-2* long isoform. We also found that, unlike *pdm-2*, the most distal 5’ UTR of the *nub* long isoform is not well conserved among different *Drosophila* species. In particular, comparative analysis revealed that the *nub* 5’ UTR is conserved in the *D. melanogaster, D. simulans, D. sechellia, D. yakuba, D. erecta, D. ananassae*, *D. persimilis, and D. pseudoobscura*, but is not present in the distantly related species.

Given the presence of the *pdm* genes in the medfly and housefly genomes, we explored whether some or all of the *Drosophila* CSCs could also be identified in these distant species. Submitting the *D. melanogaste*r genomic sequences surrounding *nub* and *pdm-2* to BLAST searches using the medfly and housefly genomes revealed sequences conserved in the three Dipteran species within several *pdm* locus CSCs (see Additional file 1: Figure S1) that were typically found within their longest conserved sequence blocks (CSBs). For example, we identified a 48 bp sequence within the **pdm2-26** CSC that is conserved in all drosophilids*,* in addition to the medfly and housefly (see Additional file 2: Figure S2).

To distinguish between adjacent CSCs, we next compared the spacing variability between CSCs in different *Drosophila* species. Previous studies show that the length of flanking less-conserved DNA sequences between adjacent CSCs varies when compared to the same regions in other *Drosophila* species [7]. These significant inter-clustal variations are due in part to species-specific insertions and/or deletions [8, 19]. In contrast, the sequence length of CSCs varies less among drosophilids. To measure the inter-clustal length differences, we identified the first and last conserved sequence in each CSC and measured the distance (in nucleotides) between CSCs in *D. melanogaster* and distant species of the melanogaster group (*D. willistoni, D. virilis, D. mojavensis and/or D. grimshaw*). Indeed, comparative genome analysis revealed significant inter-clustal variability between the *pdm* CSCs. For example, the inter-clustal distance between the CSCs **nub-14** and **nub-15** is 676 bp in *D. melanogaster*, whereas these CSCs are separated by 1458 bp in *D. mojavensis* (data not shown). To further confirm these observations, we added inter-clustal data from species closer to *D. melanogaster* and tested the statistical significance of the combined inter-clustal data using cluster analysis (see Methods). We predicted that closely related species would have similar inter-clustal values than more distantly related species. For example, inter-clustal spacing in *D. melanogaster* should more closely match spacing in *D. erecta* compared to *D. mojavensis.* We sampled the 24 CSCs upstream of the *nub* transcriptional start site in our analysis. Clustering and heatmap analysis of these CSCs (see Methods) revealed two majors species clusters: the *Drosophila melanogaster* group (*D. melanogaster*, *D. yakuba, D. erecta, D. ananassae*) and a cluster that included four outgroup species (*D. persimilis, D. pseudoobscura, D. virilis, D. mojavensis*) (Additional file 3: Figure S3). Notably, the outgroup species were clustered correctly based on their known phylogeny, which includes the *Sophophora* subgenus (*D. persimilis, D. pseudoobscura*) and the *Drosophila* subgenus (*D. virilis, D. mojavensis*) [20, 21].

### Enhancer transgene analysis reveals a wide array of *cis*-regulatory elements spanning the *pdm* locus

The *pdm* genes are expressed during multiple stages of development. For example, previous studies have shown that *nub* expression is relatively high in multiple tissues during embryogenesis and is steadily reduced in the larvae and adult, whereas *pdm-2* transcripts are detectable during embryonic and larval development [10–12]. Recent analysis of the level of expression of each of the isoforms of *nub* and *pdm-2* confirmed that RNA coding for the short and long isoforms are present both in the embryo and larvae [22]. Both of these genes are also active during multiple phases of CNS development [3, 11, 12, 23], and loss of either *pdm* gene function disrupts neurogenesis [15]. For example, in *pdm* null mutants, *cas* expression is delayed [3] and U5 motor neurons fail to form [24]. In contrast, prolonged misexpression of Pdm-2 is sufficient to activate *cas* and to produce the U5 motor neurons [24]. In addition to CNS development [3, 11], the *nub* and *pdm-2* genes are also expressed in wing imaginal discs [25] and the larval hindgut [10], respectively.

**Fig. 4:**
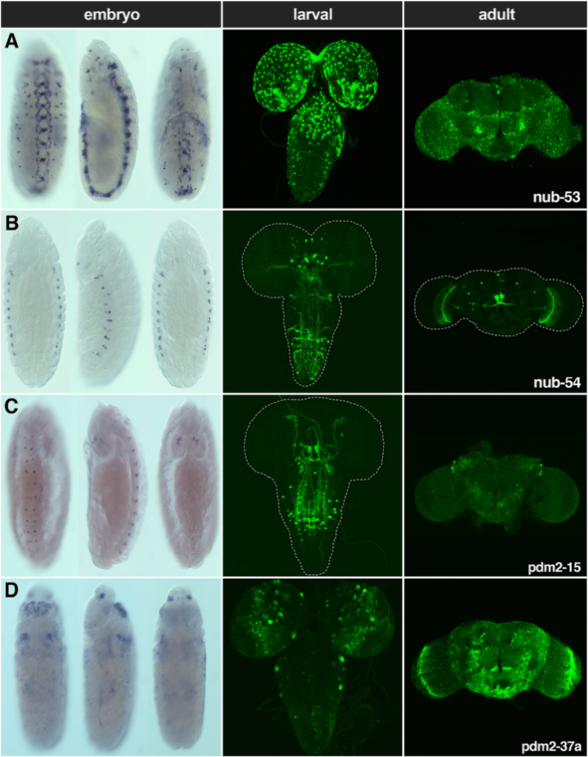
*pdm* locus enhancers direct gene expression during multiple developmental stages. **a** The nub-53 enhancer activates expression in stage 10 ventral nerve cord (VNC) cells. During postembryonic neurogenesis, nub-53 *cis*-regulatory activity in the larval brain lobes and anterior VNC was detected, as well as in the adult optic lobe and central brain. **b** nub-54 regulates expression in putative embryonic cardiac cells, larval brain lobes, and putative adult nodular neurons and median neurosecretory cells. **c** pdm2-15 enhancer activity in putative embryonic procephalon and VNC cells was detected at stage 13. pdm2-15 also directs expression in larval anteromedial and posterolateral VNC neurons and in the adult lateral horn. **d** pdm-37a activates expression in the embryonic clypeolabrum, salivary gland, and subesophageal ganglion, in larval NB lineages and throughout the adult brain

To identify neural enhancers within the *pdm* locus that activate reporter gene expression, we tested each CSC for enhancer activity during embryonic, larval, and adult neurogenesis using enhancer-reporter transgenes that were integrated into the same location on the 3rd chromosome (see Methods) [26]. Our screen identified 77 enhancers positioned throughout the *nub* and *pdm-2* noncoding sequences that generated robust reporter expression with no expression pattern variability detected among independently derived transformant lines for each of the enhancers. Reporter-gene expression patterns demonstrated that they are active either inside and/or outside of the nervous system during stages of embryonic and postembryonic development. A summary of their *cis*-regulatory activities is shown in Fig. 3 and Additional file 4: Table S1. Although a subset of these enhancers directed overlapping expression patterns, the majority activated reporter expression in unique temporal and spatial domains (Additional file 4: Table S1 and see *cis*Patterns). We also observed that many of the enhancers activated expression during multiple developmental temporal windows (Fig. 3). For example, we found that 42 enhancers drove expression in ≥ 2 developmental stages, and 13 enhancers were active in embryos, larvae and adults (Fig. 3). In particular, the **nub-53** enhancer directed expression in embryonic ventral nerve cord (VNC) cells, larval brain and VNC, and throughout the adult brain including within the central brain and optic lobe (Fig. 4a). In addition, the **nub-54** enhancer regulated expression in lateral PNS cells in the embryo, in medial brain lobe larval neurons, and in the putative nodular neurons in the adult brain (Fig. 4b). The **pdm2-15** enhancer directed expression in the embryonic procephalon, embryonic and postembryonic VNC, and adult lateral horn (Fig. 4c). The **pdm2-37a** enhancer drove expression in the embryonic clypeolabrum, salivary gland, and subesophageal ganglion, in addition to expression in the postembryonic CNS (Fig. 4d). Further studies using cell-specific markers are required to definitively identify the cell types that activate the enhancers.

To show in greater detail the *cis*-regulatory dynamics of these enhancers, we generated a web-based database that contains enhancer data collected from this survey of the *pdm* locus and from our previous studies. The website, titled *cis*Patterns (http://cispatterns.ninds.nih.gov), provides access to over 100 *Drosophila cis*-regulatory enhancers (see Methods). Information available includes images of embryonic, larval and adult expression patterns, sequence conservation, base pair length, genomic location, and keywords to facilitate searches. An online guide describes various options for viewing information. All of the GAL4 driver lines shown in *cis*Patterns are freely available to the research community.

### Embryonic expression of enhancer-reporter transgenes

**Fig. 5:**
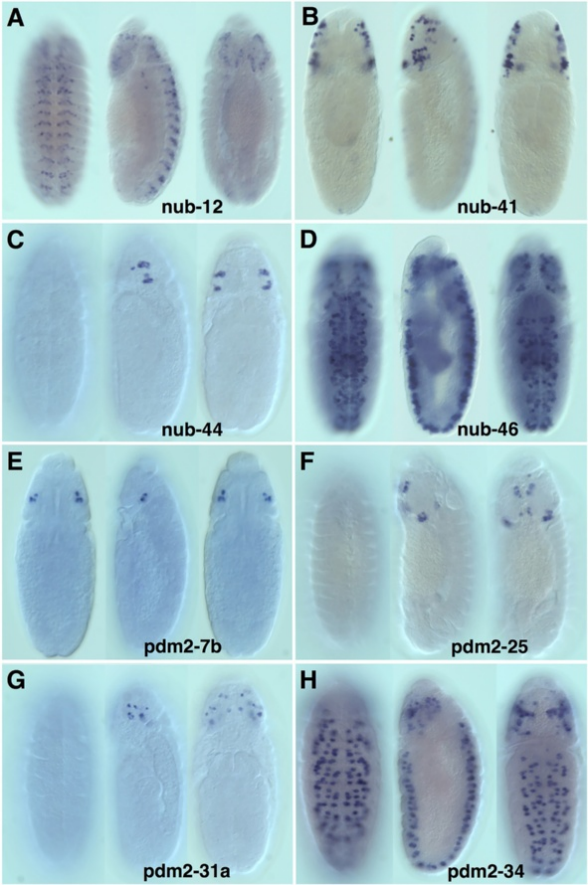
Enhancer-reporter transgene analysis reveals multiple embryonic neural-specific enhancers. Shown are 8 of the 41 embryonic enhancers that were identified in this study (see Additional file 4: Table S1 for additional embryonic enhancer descriptions). **a**–**h** Whole-mount Gal4 mRNA *in situ* hybridizations (ventral, lateral, and dorsal views); anterior up. **a** At stage 13, nub-12 directs expression in a subset of VNC and cephalic lobe neurons. **b** nub-41 regulates expression in a subgroup of lateral cephalic lobe NBs during stage 11. **c** In stage 9 embryos, nub-44 directs reporter expression in a subset of lateral cephalic lobe NBs. **d** nub-46 regulates reporter expression during stage 11 of NB lineage development in the VNC and cephalic lobes **e** pdm2-7b activates reporter expression in a limited subgroup of cephalic lobe NB during stage 10. **f** In stage 13 embryos, pdm2-25 also regulates expression in a small subset of cephalic lobe NBs. **g** pdm2-31a activates expression in cephalic lobe NBs at stage 12. **h** In stage 11 embryos, pdm2-34 directs reporter expression in subsets of VNC and cephalic lobe NBs. To definitively identify neuronal cell types, further work using neuronal-specific lineage markers is required

Among the *cis*-regulatory elements identified, we detected 41 CSCs that directed reporter expression during embryogenesis (Fig. 3 and Additional file 4: Table S1). Activity data for these enhancers is also shown at the *cis*Patterns database. Twenty-two of these enhancers activated neural expression, including the NB enhancers **nub-46** and **pdm2-34** (Fig. 5d, h, respectively). NBs were identified based on their large diameters and their position within the developing CNS. While **nub-46** and **pdm2-34** are the only enhancers that regulated expression in VNC NBs, we identified five additional enhancers that activated transgene reporter expression in cephalic lobe embryonic NBs. At stage 11, **nub-41** directed expression in a subset of NBs in the lateral region of the cephalic lobe (Fig. 5b). Similar to **nub-41**, **nub-44** also drove expression in a subset of lateral cephalic lobe NBs (Fig. 5c). However, **nub-44** regulatory activity was restricted to approximately a small subset of neural precursors per developing brain lobe at stage 9. **pdm2-7b** also regulated a very specific NB expression in the cephalic lobes at stage 10 (Fig. 5e). **pdm2-25** drove expression in posterior and medial cephalic lobe NBs at stage 13 (Fig. 5f). **pdm2-31a** directed expression in a subset of lateral and medial NBs at stage 12 (Fig. 5g). It is worth noting that the cephalic lobe NB enhancers do not appear to have overlapping regulatory activity, suggesting that these enhancers may regulate different NB sublineages during embryonic neurogenesis.

We also identified enhancers that activated expression in putative postmitotic neurons in the VNC and cephalic lobe. For example, **nub-12** was sufficient for expression in small-diameter daughters cells of NBs located in the procephalon and VNC at stage 13 (Fig. 5a). This observation is in agreement with studies that implicate a role for *nub* during asymmetric division of ganglion mother cells [27, 28].

**Fig. 6:**
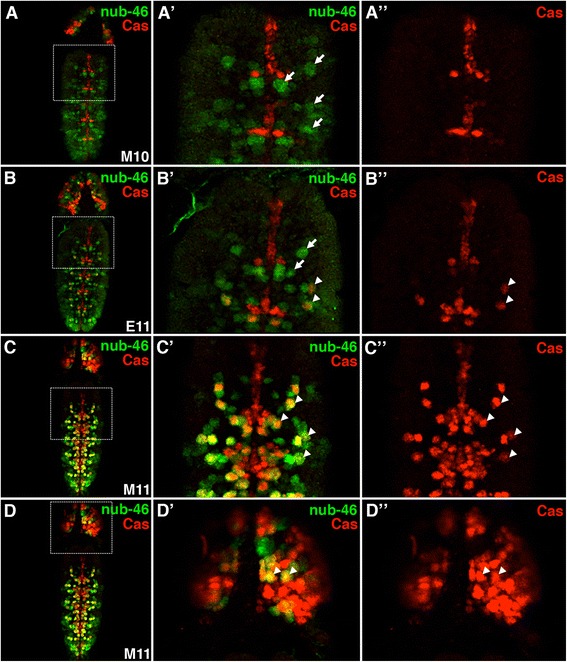
Sequential activation of the nub-46 enhancer and Castor expression during embryonic NB lineage development. **a**–**d** Shown are the dorsal views of whole-mount embryos immunostained for the presence of nub-46 enhancer activity (GFP, *green*) and Castor (*red*) expression during NB lineage development. **a** During mid stage 10 (M10), Castor is present in midline precursors in the ventral nerve cord (VNC), whereas the nub-46 enhancer regulates expression in (**a**’) a subset of Cas^−^ VNC NBs (*arrows*). The identification of the VNC NBs are based on size and location. **a**” There is no co-location of nub-46 enhancer activity and Castor expression. **b**–**b**” During early stage 11 (E11), nub-46 enhancer activity and Castor expression begins to co-localize in a subset of VNC NBs (*arrowheads*). **c**-**c**” By mid stage 11 (M11), additional VNC NBs express both Cas and the reporter gene (GFP) driven by nub-46. It is worth noting that co-localization may be in part due to perdurance of GFP. **d**–**d**” Co-localization of nub-46 activity and Cas expression is also observed in cephalic lobe cells (*arrowheads*)

We also found that enhancer activity was consistent with the temporal order of Pdm and Cas expression during NB lineage development (Fig. 6). For example, we observed the staggered onset of the initial **nub-46** enhancer activity followed by Cas expression in the developing CNS (Fig. 6). **nub-46** regulated expression in a subset of VNC NB lineages, whereas Cas was restricted to a separate group of NBs located at the VNC midline (Fig. 6a’, a”). In agreement with the transient overlap of Nub and Cas expression [3], co-localization of nub-46 enhancer activity and Cas in NBs was observed during late developmental time points (Fig. 6b–d). While the costaining of nub-46 enhancer activity and Cas shows correct temporal expression, additional work is needed to characterize the temporal window of the other NB enhancers.

### Enhancer-reporter transgene analysis during larval CNS development

We utilized the GAL4/UAS system [29, 30] to test the *cis*-regulatory potential of the CSCs during postembryonic nervous system development. To better distinguish between different cell types including NBs, GMCs and neurons, we used a membrane-bound GFP (mCD8-GFP) reporter. Our survey revealed 46 enhancers that drove expression in the larval brain and/or imaginal discs (Fig. 3). Many of the larval enhancers regulated expression in brain and VNC neurons. The following describes a subset of *pdm* enhancers that direct neuronal expression during larval CNS development and that highlight the dynamic nature of *pdm cis*-regulatory function. It is also worth noting that these enhancers are silent during embryonic neurogenesis. Expression pattern data for all 46 identified larval enhancers is provided in Additional file 4: Table S1 and shown at *cis*Patterns.

**Fig. 7:**
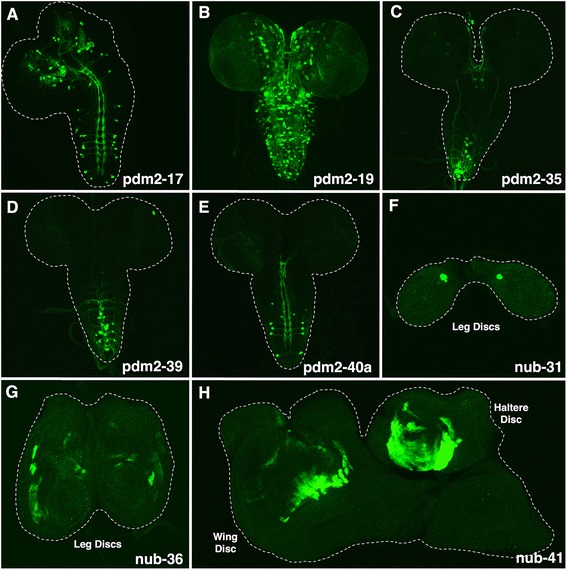
Enhancer-reporter transgene analysis reveals enhancer regulatory activity during larval CNS and imaginal disc development. Shown are the ventral views of third instar larval CNS whole-mount dissections (**a**–**e**) and whole-mount views of third instar imaginal discs (**f**–**h**). **a**–**e** Similar to *nub* conserved sequence clusters (CSCs), a subset of *pdm-2* CSCs (23 of 59 CSCs) direct mCD8-GFP expression (*green*) during larval neurogenesis (see Additional file 4: Table S1 for additional larval neural enhancers). **a** pdm2-17 directs expression in a subset of central brain and lateral VNC neurons. **b** pdm2-19 activates expression in a collection of neurons in the central brain neurons as well as lateral and medial VNC neurons. **c** pdm2-35 regulates expression in the medial central brain neurons and posteromedial VNC neurons. **d** pdm2-39 activates expression in posteromedial VNC neurons. **e** pdm2-40a directs expression in posterolateral VNC neurons. **f**–**h** A subgroup of nub CSCs (5 of 57 CSCs) activates expression in third instar larval imaginal discs including leg, wing, and haltere discs. Shown are the enhancer-reporter transgene expression patterns of nub-31, nub-36, and nub-41

**pdm2-17** regulated expression in the larval brain and VNC neurons. Enhancer activity was restricted to a narrower subset of neurons (Fig. 7a). For example, **pdm2-17** directed expression in posterior and medial anterior central brain neurons. In the VNC, enhancer activity was present in neurons with axonal projections that extend laterally to the midline, transverse, and continue longitudinally between the ventromedial and dorsomedial VNC tracts. Two pairs of these neurons are located in the third thoracic (t3) and first through fourth abdominal segments (a1-a4). Although not positively identified, their morphology and position is consistent with crustacean cardioacceleratory peptide (CCAP) neurons, which are laterally positioned in the VNC thoracic and abdominal segments [31]. These observations are also in agreement with previous studies on serotonergic lineage specification showing that *pdm* is expressed in neurons throughout the larval VNC [32]. However, additional work using specific cell markers is required to definitively determine their neuronal identity. **pdm2-19** drove expression in many brain and VNC neurons during larval neurogenesis (Fig. 7b). Unlike the **pdm2-17** pattern, GFP expression was detected in neurons within the medial and lateral central brain, as well as many neurons throughout the VNC. Notably, we did not detect any enhancer activity in the optic lobe. **pdm2-35** drove expression along the midline of the larval brain and VNC (Fig. 7c). Reporter expression was restricted to a single neuron in each anterior medial brain lobe, a pair of symmetric neurons in the medial thoracic 1 (t1) and thoracic 2 (t2) segments, and a subset of midline neurons in the lower abdominal VNC. **pdm2-39** is located ~3.5 kb downstream of **pdm2-35** and also drove expression in midline neurons (Fig. 7d). Further, we observed enhancer activity in dorsolateral neurons in the fifth through seventh abdominal (a5-a7) segments. Their axonal projections cross and then ascend the midline of the VNC. **pdm2-40a** is immediately adjacent to **pdm2-39** but regulated expression in a different subset of VNC neurons (Fig. 7e). The expression pattern was made up of bilateral pairs of neurons located in the lateral t3, medial a1, lateral a2-a4 and lateral a7 segments. Given their location and morphology, the lateral a2-a4 cells may be serotonergic neurons. Consistent with this prediction is that lateral serotonergic neurons express *pdm* during larval neurogenesis [23].

We also identified enhancers that activated reporter expression in imaginal discs. These enhancers (**nub-19**, **nub-31**, **nub-32b**, **nub-36**, and **nub-41**) were active in different imaginal discs (described in Additional file 4: Table S1 and shown at *cis*Patterns). The identification of disc enhancers is in agreement with previous studies that have detected Nub expression in wing discs via immunostaining [25, 33]. Of the five disc enhancers, **nub-31** drove expression in a subset of cells occupying the dorsal anterior region of the leg imaginal disc [34] (Fig. 7f). **nub-36** directed weak expression in the leg imaginal disc (Fig. 7g). Specifically, the expression overlapped a region that develops into the coxa, an adult appendage connecting the leg to the thorax [35]. **nub-41** drove expression in both the haltere and wing imaginal disc. However, compared to **nub-31** and **nub-36**, there was no leg disc expression. In the haltere (also referred to as the rudimentary wing) disc, the expression pattern was composed of cells that develop into the pedicel and capitellum segments of the adult rudimentary wing based on their location [36] (Fig. 7h). In the wing disc, the enhancer regulated expression in cells that will become part of the proximal wing [35] (Fig. 7h).

**Fig. 8:**
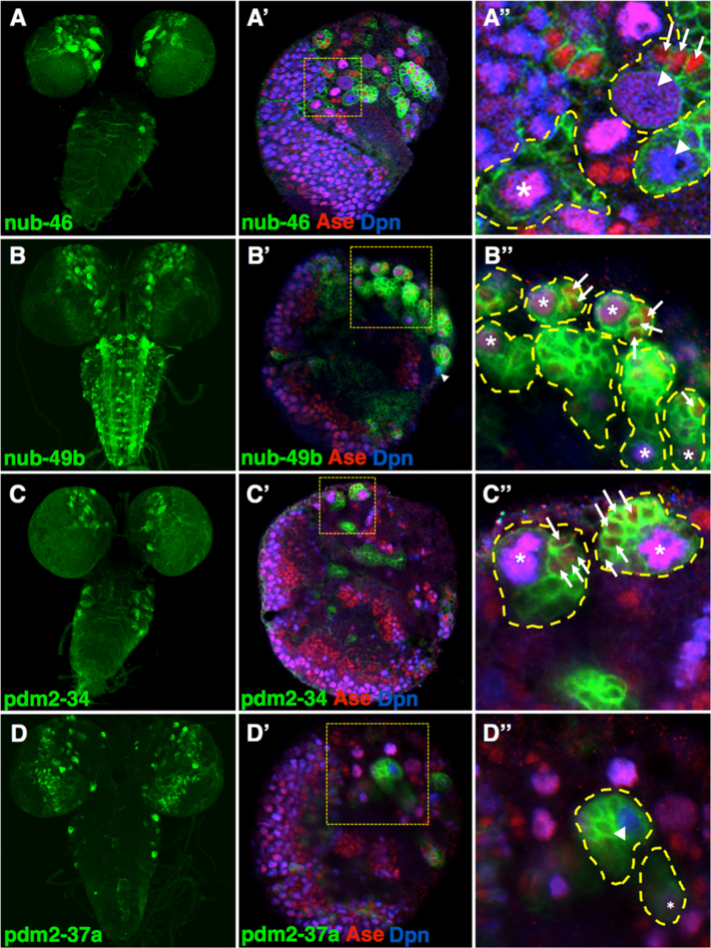
*pdm* locus enhancers drives expression in specific classes of larval NBs. **a**–**d** *pdm* enhancers activate reporter expression during larval NB lineage development. Shown is membrane-bound GFP (mCD8-GFP) expression (*green*) driven by each enhancer. **a** The nub-46 enhancer regulates central brain and VNC expression. **a**’ The nub-46 enhancer regulates expression in type I and type II NBs. Shown is a single confocal plane view of a larval brain lobe stained with anti-GFP (*green*), anti-Ase (*red*), and anti-Dpn (*blue*). **a**” The inset is a magnified view of the yellow dashed square and highlights type I NBs (Ase^+^ Dpn^+^, *asterisk*), type II NBs (Ase^−^ Dpn^+^, *arrowheads*), GMCs (Ase^+^ Dpn^−^, *arrows*) and individual NB lineages (*yellow dashed outlines*). **b** The nub-49b enhancer regulates a subset of optic lobe, central brain, and VNC NB lineages. **b**’ and **b**” show that nub-49b directs expression in type I and type II NBs. **c** The pdm2-34 enhancer directs central brain and VNC expression. **c**’ and **c**” reveals that pdm2-34 drives expression in type I NBs. **d**–**d**” The pdm-37a enhancer activates expression in type I and type II larval NBs

A subset of enhancers drove expression in putative larval NBs. For example, the two embryonic NB enhancers for *nub* and *pdm-2*, **nub-46** and **pdm2-34,** are also active in larval NBs (Fig. 8a, c, respectively). In addition, **nub-49b** and **pdm2-37a** also regulated expression during larval NB lineage development (Fig. 8b, d).

As a first step toward identifying the NBs that activate the *pdm* enhancers, we carried out co-localization studies with type I and type II NB markers. Previous work by others has identified two types of larval NBs that differ in their cellular division and renewal [37]. Similar to embryonic NBs, type I NBs divide asymmetrically to produce several ganglion mother cells (GMCs), each of which undergo a single round of division to form two progeny. In contrast, type II NBs first produce a NB-like cell called an intermediate neural progenitor (INP) which then divides asymmetrically to create GMCs. Differences between type I and type II NBs suggests that distinct expression programs may regulate their cellular identities. Indeed, type I NBs express the TFs Deadpan (Dpn) and Asense (Ase), whereas only Dpn is detected in type II NBs [37]. Our coexpression studies using the Dpn and Ase markers revealed that **nub-46** (Fig. 8a’, a”), **nub-49b** (Fig. 8b’, b”), and **pdm2-37a** (Fig. 8d’, d”) drove expression in type I and type II NBs. In contrast, **pdm2-34** enhancer activity was detected only in type I NBs (Fig. 8c’, c”).

### Enhancer-reporter transgene analysis in the adult brain

**Fig. 9:**
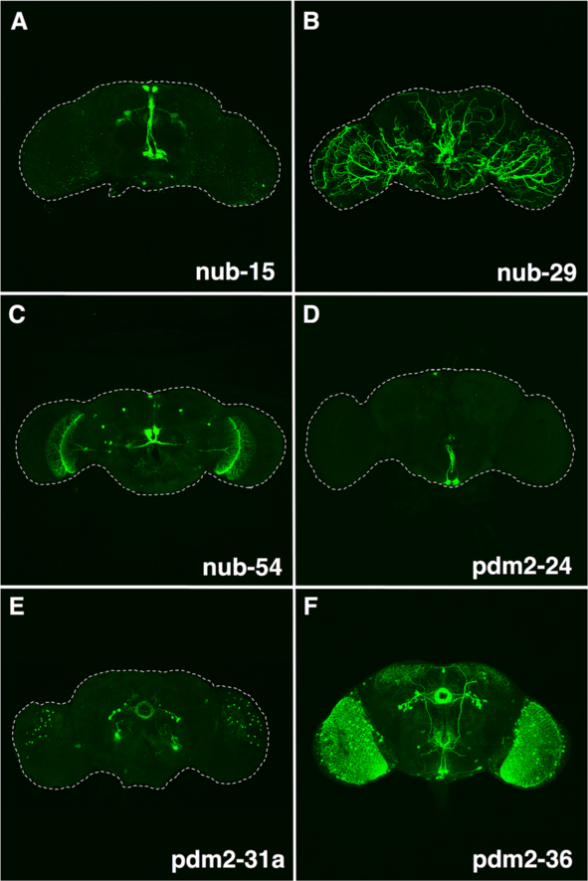
Analysis of enhancer-reporter transgenes in the adult brain identifies both neural and tracheal enhancers. **a**–**f** Shown are 6 of the 46 *pdm* locus enhancers that direct mCD8-GFP expression (*green*) using the GAL4/UAS system in adult brains (see Additional file 4: Table S1 for additional adult enhancers). **a** nub-15 regulates expression in putative median neurosecretory cells, ventrolateral protocerebrum, ellipsoid body, lateral triangle, and cell body. **b** nub-29 directs expression in the adult tracheal branches. **c** nub-54 regulates expression in noduli and median neurosecretory cells. **d** pdm2-24 regulates expression in a subset of cells in the subesophageal ganglion. **e** pdm2-31a directs expression in the ellipsoid body, cell body, medulla, and ventromedial protocerebrum. **f** pdm2-36 activates expression in lobula, optic glomerulus, medulla, subesophageal ganglion, ellipsoid body, cell body, lateral triangle, and median neurosecretory cells. Note: the above expression pattern descriptions are based on previous work that defines adult neuroanatomy

Our survey revealed 46 enhancers that drove expression within the adult brain and each activated expression in overlapping patterns (see Additional file 4: Table S1 and shown at *cis*Patterns). For example, twenty-five enhancers directed expression in putative median neurosecretory cells (mNSCs) based on their previously described distinct morphology and location (see Additional file 4: Table S1) [38–40]. mNSCs are located in the superior medial protocerebrum and send their projections to a *Drosophila* gustatory system known as the tritocerebrum – reflecting the cellular morphology that overlaps enhancer activity. **nub-15** is a 1.2 kb enhancer that activates expression in putative mNSCs (Fig. 9a). We also detected expression in putative central complex neurons, which play a role in locomotion, vision, learning and memory [41]. The **nub-15** enhancer regulated expression in the ellipsoid body, lateral triangle and cell body – three structures readily identifiable according to their position and morphology [41]. Twenty-three enhancers were identified that drove neuronal expression in the subesophageal ganglion (SOG), another gustatory center located in the most ventral region of the central brain (see Additional file 4: Table S1) [40]. Unlike a majority of the SOG enhancers that directed broad expression, **pdm2-24** enhancer activity was limited to a single symmetric pair of putative SOG neurons (Fig. 9d).

Interestingly, we identified 22 enhancers that regulated neuronal expression in both mNSCs and SOG neurons, albeit in non-identical subsets of SOG neurons (see Additional file 4: Table S1 and *cis*Patterns). For example, **pdm2-36** directed expression in mNSCs and a symmetric medial pair of SOG cells (Fig. 9f). These SOG neurons ascend to the tritrocerebrum, decussate, and form dense axonal arborizations in the dorsolateral protocerebrum. In addition, we detected **pdm2-36** enhancer activity in optic lobe structures, including the lobula plate, optic glomerulus and medulla. Further, similar to the **nub-15** enhancer, **pdm2-36** drove expression in cells of the central complex; namely, the ellipsoid body, lateral triangle and cell body.

The screen also identified other *cis*-acting elements that directed expression in the central complex. **pdm2-31a** is located 6 kb upstream of **pdm2-36** and was also sufficient for expression in the ellipsoid body and cell body (Fig. 9e). We observed **pdm2-31a** enhancer activity in putative ventromedial protocerebrum and medulla neurons. **nub-54** was largely restricted to a pair of putative central complex neuropils termed noduli (Fig. 9c) [42]. Previous work has indicated that noduli are connected to neural circuitry for visual processing in insects [39]. This is consistent with our findings showing that axonal projections of these neuropils decussate immediately at the dorsal side of the esophagus and continue laterally to innervate the lobula plate in the optic lobe (Fig. 9c). We have also identified enhancers that drive non-neural expression in the adult. For example, **nub-29** regulated expression in both the optic lobe and central brain tracheal branches (Fig. 9b).

### Comparative sequence analysis reveals unique combinations of shared conserved elements among functionally related enhancers

**Fig. 10:**
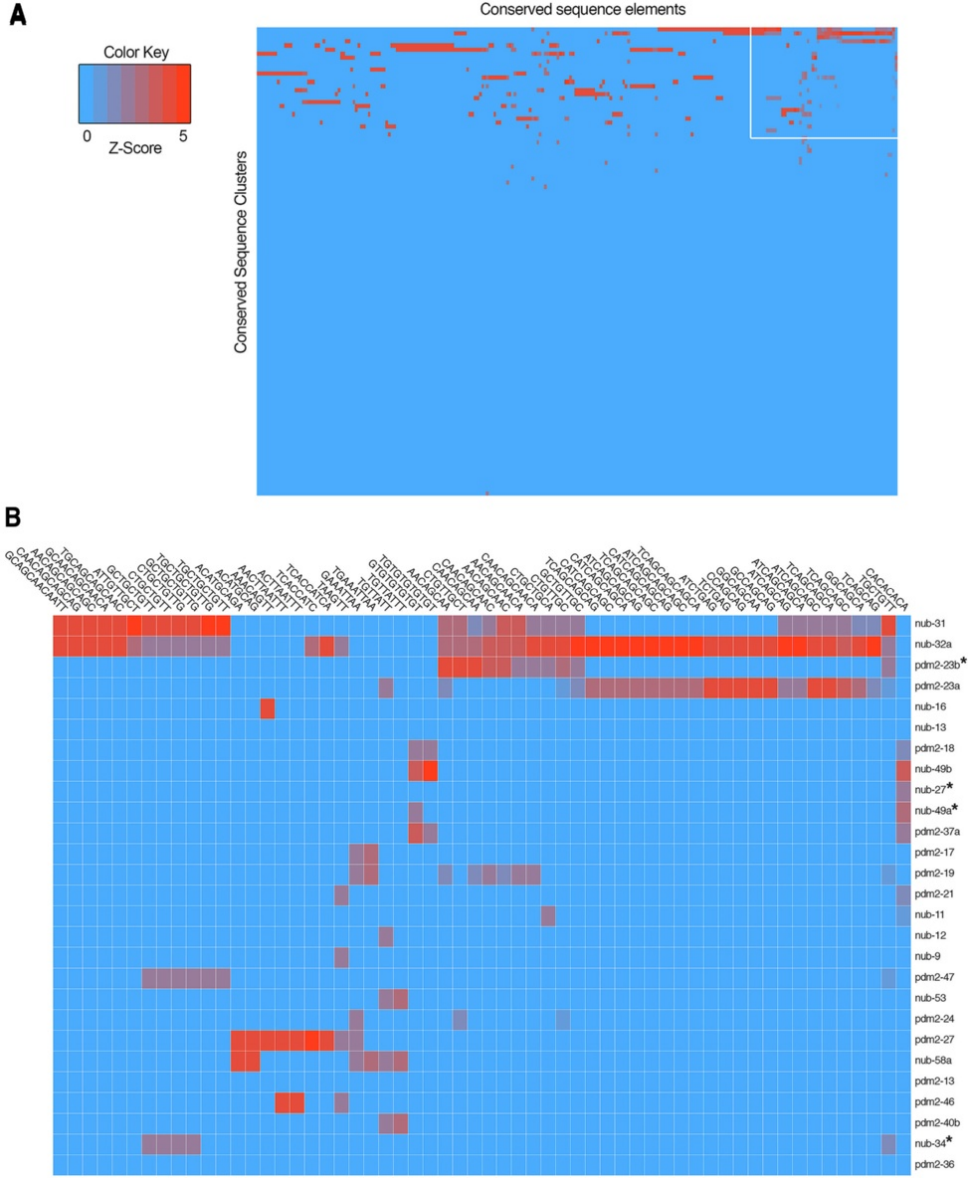
Comparative analysis of conserved sequences within *pdm* locus enhancers reveals elements shared among SOG enhancers. **a** Shown is a heat map representation of conserved sequence elements extracted from *pdm* SOG enhancers and compared to all *pdm* locus conserved sequence clusters (see Additional file 4: Table S1) using hierarchical clustering among shared conserved elements. Data was normalized to generate standardized scores (Z-scores) illustrated by the color key. The white outlined area represents panel (**b**), an enlarged portion showing that the 23 *pdm* SOG enhancers cluster based on these conserved elements. Columns represent shared conserved elements, whereas *pdm* locus conserved sequence clusters are shown in rows. It is also worth mentioning that the group also contains false positives, or non-SOG conserved sequence clusters (*asterisks*). Note: a more detailed description of the heat map is available upon request

To investigate whether the functionally related enhancers discovered in this study can be classified based on their shared conserved sequence elements, we compared the 23 enhancers that drove neuronal expression in the SOG of adult *Drosophila*. To determine if they share conserved sequences, we developed a computational method to handle all pairwise combinations of these enhancers. We also assessed whether conserved elements shared among the SOG enhancers were also found in *pdm* locus CSCs that did not activate reporter expression in SOGs. To accomplish this, we generated a library of shared conserved sequence elements within SOG enhancers, measured the frequency of these elements within SOG and non-SOG CSCs, and preprocessed this information for elements occurring predominantly in SOG enhancers. This approach returned 254 unique conserved DNA elements, the length of each n-mer ranging from 6 to 12 bp (Additional file 5: Table S2). Hierarchical cluster analysis revealed that these shared elements are sufficient to distinguish the SOG enhancers from other *pdm* locus CSCs (Fig. 10). Interestingly, we also observed that each CSC contained a different combination of these conserved sequence elements. For example, **nub-31** and **nub-32a** contain 68 and 71 of the 254 conserved DNA elements, respectively, but only share 41 of these conserved elements. We also identified quantitative differences among the shared their elements. While the two enhancers both contain the conserved DNA sequence TGCTGCTGTTG, the 11-mer is present twice in **nub-31** and once in **nub-32a**. It is worth noting that we identified 4 of the 93 non-SOG CSCs (**nub-27**, **nub-34**, **nub-49a**, and **pdm2-23b**) clustered within the SOG enhancer group (Fig. 10b, asterisks). We next determined whether this approach could group other functionally related *pdm* enhancers. As previously mentioned, we identified 25 enhancers that drove expression in adult median neurosecretory cells. Similar to the SOG enhancers, these *cis*-regulatory modules clustered together in the hierarchical clustering analysis based on their uniquely shared conserved sequence elements that are largely absent in non-mNSC CSCs within the *pdm* locus (data not shown). Further comparative analysis is required to enhance resolution of this comparative method. Nevertheless, these common elements suggest a possible shared combinatorial nature within functionally related enhancers.

Structurally similar sets of neural enhancers were also found tandemly arrayed in multiple locations within the *pdm* locus (Fig. 3). For example, the consecutively arrayed enhancers **pdm2-17**, **pdm2-18**, and **pdm2-19** drove overlapping neural expression patterns in larvae and adults. As described above, **pdm2-17** and **pdm2-19** regulated expression in larval brain and VNC neurons (Fig. 7a, b). Similarly, the enhancer activity of **pdm2-18** was detected in brain and VNC neurons and is similar to **pdm2-19** enhancer function, albeit, in fewer medial VNC neurons and in a greater number of lateral VNC neurons (see Additional file 4: Table S1 and *cis*Patterns). Another example of adjacent enhancers spans ~6 kb of noncoding DNA and is located ~22.5 kb upstream to the *nub* long isoform. This array contains 6 CSCs: **nub-8** thru **nub-13** (Fig. 2). Regulatory activity differed among these enhancers in the three tested developmental phases (Fig. 3). For example, we detected embryonic enhancer activity for **nub-9**, **nub-11**, and **nub-12**, whereas **nub-8**, **nub-10**, and **nub-13** did not activate reporter expression during embryogenesis. In the adult brain, all enhancers drove CNS expression except for **nub-10**. Further, these enhancers are active and have overlapping function during larval CNS development. In particular, each enhancer regulated expression in lateral VNC neurons (Additional file 6: Figure S4); however, each enhancer directed expression in a different number of lateral VNC neurons. For example, **nub-9** drove reporter expression in most but not all lateral neurons in every thoracic and abdominal VNC segment (Additional file 6: Figure S4B), whereas **nub-8** enhancer activity was restricted to markedly fewer cells in comparison (Additional file 6: Figure S4A).

**Fig. 11:**
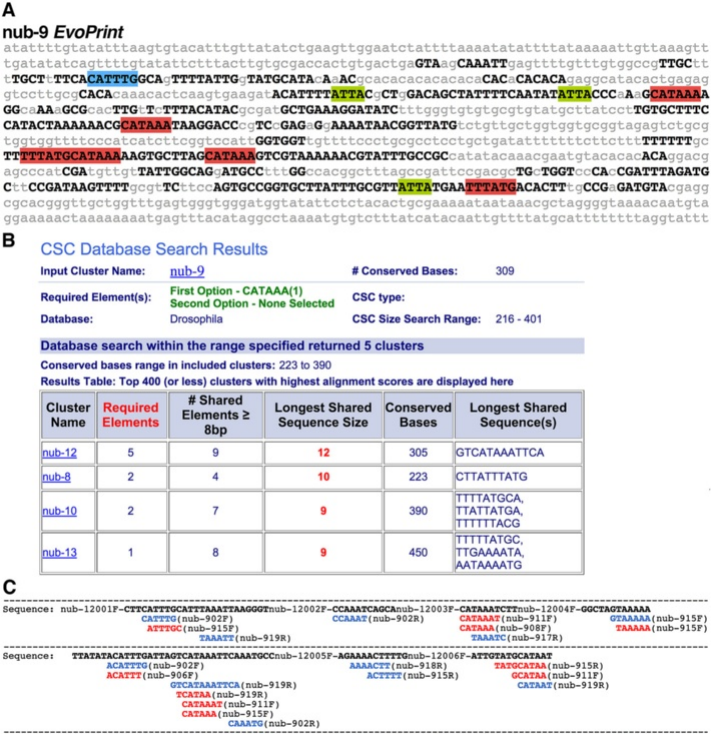
Pair-wise alignments of *nubbin* neural enhancer sequences reveal that many share conserved sequence elements. **a** Phylogenetic comparative analysis of nub-9 enhancer identifies multiple conserved sequence blocks. *Black capital letters* represent *D. melanogaster* bases conserved in *D. simulans, D. sechellia, D. yakuba, D. erecta, D. ananassae, D. persimilis, D. pseudoobscura, D. willistoni, D. virilis, D. mojavensis and D. grimshaw* orthologous DNA sequences. *Lowercase gray letters* represent bases not conserved in one or more species included in the analysis. Putative TF DNA-binding sequences are highlighted as follows: Castor/Hunchback DNA-binding motifs (CATAAA/TTTATG) are *red highlighted* sequences; bHLH DNA-binding sites (CANNTG) are *blue highlighted* sequences; and Hox DNA-binding motifs (ATTA/TAAT) are *green highlighted* sequences. **b** Advanced *cis*-Decoder search results of nub-9 for a subset of *nub* conserved sequence clusters (CSCs) that each contain the conserved 5’ CATAAA 3’ element. For each CSC aligned with the input nub-9 *EvoPrint*, the results table provides the following statistics: the number of required elements present in the CSC; number of shared elements ≥8 bp; the longest shared sequence length (sequences in *red* indicate that it contains the required sequence, CATAAA); total number of conserved bases; and the longest shared sequence(s). An example of an alignment is shown in panel (**b**). **c** Shown is a *cis*-Decoder alignment of nub-9 and nub-12 conserved sequences that highlights ≥6 bp shared elements. The readout indicates the nub-9 single copy (*blue*) and repeated (*red*) conserved sequences aligned to nub-12 CSBs (*bold black*). Displayed in 5’ to 3’ order, CSBs are annotated to reflect their ordered appearance within the enhancers and their alignment orientation (forward, F; reverse, R)

To determine whether these functionally related enhancers share sequence motifs, we then performed a pairwise comparative analysis to identify shared conserved sequences. We employed a specialized feature of *cis*-Decoder called *Advanced Search* that computes a pairwise alignment between a reference CSC and a user-generated library of CSCs. For this, we chose **nub-9** (Fig. 11a) as the reference CSC and added the remaining five CSCs to a library. Our comparative analysis revealed that the CSCs share many conserved sequences. For example, all of the CSCs except **nub-11** contain several copies of the 6-mer CATAAA that corresponds to the DNA-binding site for Hb [13, 14] and Cas [3]. In particular, the putative Hb/Cas docking site was detected multiple times within conserved sequences in **nub-8** (2 sites), **nub-9** (6 sites), **nub-10** (2 sites), and **nub-12** (5 sites) (Fig. 11b, c). Only **nub-13** has a single but extended Hunchback/Castor DNA-binding motif (CATAAAAAA/TTTTTATG, Fig. 10b), which has a greater similarity to the consensus sequence [3, 13, 14] than does the 6-mer.

## Discussion

Our analysis of the *pdm cis*-regulation indicates that the spatiotemporal dynamics of their expression is controlled by a functionally diverse array of modular enhancers. Analysis of the 125 kb *pdm* locus identified 77 *cis*-regulatory enhancers that activate gene expression in the embryo, larvae and/or adult. Our studies also revealed that many of the functionally related neural enhancers that direct overlapping expression patterns are tandemly arrayed. We found 41 enhancers directed embryonic expression, an overlapping set of 46 activated larval expression, and another overlapping set of 46 activated expression in the adult CNS. While many of these enhancers were activated only in the nervous system, a subset activated reporter gene expression outside of the nervous system, including in larval appendages and in the trachea. Roughly a third of the tested CSCs did not exhibit any detectable *cis*-regulatory activity in the nervous system. Since our focused on identifying neural enhancers, the possibility exists that some or all of these CSCs that lack neural system activity may regulated gene expression in the larval and adult tissues that were not examined.

There are other online resources of documented enhancers in the *Drosophila* genome, namely, FlyLight [43–46] and Vienna Tiles [47]. While these *cis*-regulatory libraries provide useful information, the coverage of the *pdm* locus in these databases is not complete. For example, FlyLight analysis did not detect 14 enhancers that flank the *nub* transcribed sequence. These include those located upstream to the *nub* long transcript (**nub-12** and **nub-13**), its first intron (**nub-28**), second exon (**nub-32a**), second intron (**nub-32b**, **nub-32c**, **nub-33**, **nub-36**, **nub-40b**, **nub-41**, **nub-42**, **nub-44**, and **nub-45a**), and third intron (**nub-49b**) (Fig. 3). The FlyLight library also does not include seven *pdm-2* enhancers: located in the upstream region (**pdm2-21**); within the second intron (**pdm2-27** and **pdm2-28**) and lacks information regarding its downstream region (**pdm2-45**, **pdm2-46**, **pdm2-47** and **pdm2-48**) (Fig. 3). Vienna Tiles also provides only partial coverage of the *pdm* locus, omitting the following 11 *pdm* locus enhancers: **nub-58a**, **nub-58b**, **pdm2-13**, **pdm2-17**, **pdm2-21**, **pdm2-22**, **pdm2-23a**, **pdm2-31b**, **pdm2-32**, **pdm-33**, and **pdm2-48** (Fig. 3). While the Vienna Tiles database provides information on embryonic and adult enhancers, it does not supply information on *cis*-regulatory activity during larval development. In addition, based on our analysis, most of the reporter transgenes in these two libraries contain multiple enhancers. For example, we observed that the Vienna Tiles enhancer denoted as VT6436 enhancer is made up of two embryonic enhancers (**nub-28** and **nub-29**).

Analysis of the *pdm* locus enhancers identified four functionally related enhancers (**nub-46**, **nub-49b**, **pdm2-34**, and **pdm2-37a**) that activated expression during NB lineage development. The **nub-46** and **pdm2-34** enhancers are both located in the third intron of the *nub* and *pdm-2* long transcript, respectively, whereas **nub-49b** and **pdm2-37a** are positioned immediately 5’ to the transcriptional start site of their respective short isoform (Additional file 7: Figure S5). While the **nub-46** and **pdm2-34** enhancers drove overlapping but nonidentical expression during embryonic and larval NB lineage development (Fig. 5d, h, Fig. 8a, c), **nub-49b** and **pdm2-37a** regulated similar expression patterns during postembryonic NB lineage development (Fig. 8b, d). Analysis of **nub-46** and **pdm2-34** revealed that these enhancers share multiple conserved DNA elements, albeit in largely unique configurations (data not shown). Although these observations suggest these enhancers are related, additional studies are needed to further resolve subtle differences between their regulatory activities.

Comparative analysis of the *nub and pdm-2* coding sequences revealed that their sequence relationship was mostly limited to the exons that encode their POU domains and homeodomains. In contrast, we did not detect any evidence of collinearity within their noncoding regions, suggesting that they have diverged at a faster rate than the coding sequences. We also identified only one *pdm* ortholog in the mosquito, whereas the medfly and housefly carry both genes. Given this observation and accounting for the divergence of *Drosophila* from these distant Diptera [16, 17], the *pdm* duplication event may have occurred in the Dipteran line between 100 and 260 million years ago.

Despite the lack of sequence relationship within the noncoding sequences, the *pdm* locus is enriched with clusters of conserved sequences and some of them have been maintained in other Diptera. Our studies revealed that two-thirds of the CSCs function as *cis*-regulatory enhancers that regulate gene expression in a diverse array of spatiotemporal aspects, which taken together reflect *pdm* expression domains. These observations suggest that the *pdm* genes are dynamically regulated by multiple *cis*-regulatory modules, and that these enhancers are more amenable to evolutionary restructuring than their protein encoding exons. This is in agreement with recent reviews on the evolution of Dipteran enhancers highlighting the flexibility of enhancers to maintain their function after loss and/or gain of TF DNA binding sites [48, 49]. Also consistent with these observations, we discovered functionally related enhancers within the *pdm* locus that share conserved sequences, albeit in different arrangements and orientations.

From a mechanistic perspective, our observations suggest that enhancer behavior can be predicted based on the combination of the conserved elements shared among functionally related enhancers. Similar observations have been made by Aerts and Schweisguth laboratories [50, 51]. Hierarchical clustering analysis of shared conserved sequences revealed that *pdm* SOG enhancers may be grouped based on shared elements that are for the most part not present within other *pdm* locus CSCs. A similar analysis of adult median neurosecretory cell (mNSC) enhancers revealed that they grouped together, as evidenced by sharing of conserved sequence elements, which were largely absent in non-mNSC CSCs with the *pdm* locus. While further work is required to determine whether these shared elements are important for enhancer activity, these findings suggest a level of structural complexity in the presence and clustering of enhancers that requires further analysis. To construct a better representation of enhancer structure and thus *cis*-regulatory prediction, one would ideally prefer to use a larger training set of enhancers to improve the accuracy of prediction [52]. These approaches will be addressed in future studies.

## Conclusions

One of the principal findings of this study is the discovery of 77 enhancers that exhibit a remarkably diverse range of *cis*-regulatory activities during embryonic and postembryonic development. The biological significance of this enhancer diversity most likely reflects the diversity of the developmental programs in which these transcription factors participate. We also identified functionally related enhancers that share multiple conserved DNA sequences and determined that these enhancers could be classified using hierarchical clustering techniques. In addition, our analysis has revealed that the collinearity between the *pdm* genes is predominantly confined to their POU domain and homeodomain exons, suggesting that their noncoding sequences are diverging at a faster rate than their coding sequences. These results should provide further insight into the regulatory logic that controls *cis*-regulatory function and thus gene regulation.

## Methods

### Comparative genomics

The UCSC Genome Browser was used to retrieve DNA sequences within the *pdm* locus (http://genome.ucsc.edu/). The *pdm* locus is approximately 125 kb (chr2L:12,565,558-12,690,307). The phylogenetic comparative analysis of these sequences was performed using the *EvoPrinter* programs (http://evoprinter.ninds.nih.gov/) and included the 12 available drosophilids. CSCs identified from overlapping *EvoPrints* were annotated to include gene name hyphenated with consecutive numbers and were named based on their proximity to the *nub* and *pdm* genes. Pairwise alignments of these CSCs were performed using the *cis*-Decoder program (http://cisdecoder.ninds.nih.gov). Instructions for both *EvoPrinter* and *cis*-Decoder are provided on their respective websites.

### Hierarchal clustering and heat map analysis

We sampled inter-clustal spacing variability between 24 CSCs upstream of the *nub* long transcript in 8 drosophilids, including *D. melanogaster*, *D. yakuba*, *D. erecta*, *D. ananassae*, *D. persimilis*, *D. pseudoobscura*, *D. virilis*, and *D. mojavensis*. The inter-clustal spacing values for each species were stored in a data matrix file. Hierarchal clustering and heat map analysis were performed using R, a statistical programming language environment (http://www.r-project.org/). We employed the *gplots* package that includes *heatmap.2*, the hierarchal clustering and heat map algorithm. We employed a similar protocol to determine shared conserved DNA elements in functionally related *pdm* enhancers. Using parsing algorithms, we extracted conserved DNA elements (6- to 12-mers) from SOG enhancers identified within the *pdm* locus and measured their occurrence within 23 SOG and 93 non-SOG *pdm* enhancers. We further screened for conserved elements with relatively high frequency within SOG enhancers and performed hierarchical clustering using this data set after normalization. The same approach was used to analyze the mNSC enhancers. All algorithms are available upon request.

### Enhancer-reporter transgene constructs

A modified pCa4B vector was employed in these studies [8]. The pCa4B vector was modified to include the following features from the pHStinger vector [53]: the pHStinger polylinker (replacing the pCa4B polylinker), a minimal Heat shock protein 70 (Hsp70) promoter driving a GAL4 or GFP reporter gene, and gypsy chromatin insulators to block influence of flanking enhancers that would otherwise modify reporter expression via enhancer trap effects. The vector also contains bacterial attachment (attB) sites for its targeted chromosomal insertion [26]. The site-specific integration vector was selected to ensure that all of the enhancer-reporter constructs were inserted in the same chromosomal environment. In addition to the gypsy chromatin insulators, the nonrandom integration afforded by the f31 integration further reduces integration variability on enhancer function. Integration of the pCa4B vector is facilitated by a serine integrase, phage f31, which mediates recombination between vector attB sites and genomic phage attachment (attP) sites [26].

### Generation of transgenic fly lines

CSC-containing DNA fragments were cloned from wild-type genomic DNA using standard PCR methods. PCR products were analyzed using gel electrophoresis and were purified by a Qiagen QIAquick Gel Extraction Kit. Purified PCR products were inserted into the Invitrogen pCRII-TOPO TA vectors. Plasmids with CSC-containing DNA fragments were sequenced by the NIH DNA Sequencing Core Facility to confirm their sequences. Verified sequences were inserted into the modified pCa4B vector described above in the *Enhancer-Reporter Transgene Constructs* section. Construct DNA were injected into attP2 (insertion site on chromosome 3L, 68A4) [54] fly embryos by Rainbow Transgenic Flies, Inc. and independent transformant lines for each construct were generated. Standard genetic crosses were performed to generate homozygous transgenic fly lines. Fly lines are maintained at 18 °C using standard husbandry procedures [55].

### Immunohistochemistry

 Embryo collections and fixations of transgenic fly lines were performed according to procedures previously described [56]. For *in situ* hybridizations, mRNA probes were generated from a PCR amplified GAL4 or GFP ORF. All *pdm* locus enhancers directed GAL4 reporter expression, except for nub-19, nub-53, and pdm2-8a, which was detected by enhancer-GFP expression. Roche DIG RNA Labeling Mix protocol was used and staining was visualized using anti-FITC Fab fragments coupled to alkaline phosphatase (1:2000, Roche). After whole-mount *in situ* hybridization, embryos were photographed using a Nikon Optiphot microscope (10X objective lens). Embryo developmental stages were determined based on morphological features previously described [57]. In addition, larval and adult brains were dissected and fixed according to protocols previously outlined [58]. For single immunolabeling, purified rabbit anti-GFP (1:1000, Invitrogen) and anti-rabbit Alexa 488 (1:000, Invitrogen) were used.

For triple immunolabeling of larval NBs, primary antibodies mouse anti-GFP (1:1000, Chemicon), rabbit anti-Asense (1:1000, gift from Tzumin Lee), guinea pig anti-Deadpan (1:500, gift from James Skeath) were used, together with anti-mouse Alexa 488 (1:1000, Invitrogen), anti-rabbit Alexa 568 (1:1000, Invitrogen), and anti-guinea pig Alexa 633 (1:1000, Invitrogen). For Castor co-staining, primary chicken anti-GFP (1:500, Chemicon) and rabbit anti-Castor (1:500) antibodies were used together with anti-chicken Alexa 488 (1:1000, Invitrogen), and anti-rabbit Alexa 633 (1:1000, Invitrogen) secondary antibodies. Fluorescence whole-mount immunolabeling techniques were carried out according to procedures previously described [58]. After immunolabeling, third instar larval and adult CNS tissue were examined for GFP expression. Serial optical sections of dissected brains were photographed at 1 mm intervals using a Zeiss LSM 510 confocal microscope (10× objective lens, 40× objective lens for single larval brain lobes).

### cisPatterns algorithms and database

The *cis*Patterns program is installed on NINDS servers. The algorithms for the *cis*Patterns user interface was developed using standard techniques used in the HTML (HyperText Markup Language), PHP (PHP: Hypertext Preprocessor), and JavaScript web programming languages [59].

### Availability of supporting data

The results of our analysis are catalogued in *cis*Patterns (cispatterns.ninds.nih.gov).

## Additional files

Additional file 1: Figure S1. Three-way alignment of ultraconserved sequences in conserved sequence clusters identified in Drosophila, housefly, and medfly. Shown are conserved sequences shared in *Drosophila* conserved sequence clusters (nub-35, nub-37, pdm2-6, pdm2-12, and pdm2-21) and detected in the housefly (*Musca domestica*) and medfly (*Ceratitis capitata*). Vertical lines indicate agreement among all three Diptera. (TIFF 6544 kb) Additional file 2: Figure S2. The pdm2-26 enhancer contains ultraconserved sequences detected in multiple Diptera. A) Shown is a 12-drosophilid *EvoPrin*t of the pdm2-26 enhancer sequence together with colored highlights that indicate conserved *D. melanogaster* sequences shared with both the housefly and medfly (*Musca domestica* and *Ceratitis capitata*, respectively; orange) or with the medfly only (blue). Black capital letters represent *D. melanogaster* bases conserved in *D. simulans, D. sechellia, D. yakuba, D. erecta, D. ananassae, D. persimilis, D. pseudoobscura, D. virilis,* and *D. mojavensis*. **B)** and **C)** Raw BLAST results of the pdm2-26 conserved elements aligned to the housefly (*Musca domesticus*) and medfly (*Ceratitis capitata*) genomes, respectively. The colored underlines correspond to the colored highlighted conserved sequences in panel **A)**. (TIFF 7785 kb) Additional file 3: Figure S3. Resolution of conserved sequence clusters is enhanced by evolutionary flexibility in their flanking less-conserved DNA. Shown is a dendrogram tree that illustrates the similarity among Drosophila species based on the inter-clustal spacing of their conserved sequence clusters (CSCs). This also includes 24 consecutive CSCs across 8 *Drosophila* species (*D. melanogaster*, Mel; *D. erecta*, Erec; *D. yakuba*, Yak; *D. ananassae*, Ana; *D. mojavensis*, Moj; *D. virilis*, Vir; *D. persimilis*, Per; and *D. pseudoobscura*, Pse). The heat map represents a visualization of inter-clustal spacing flexibility, where blue and red blocks highlight lower and higher inter-clustal spacing flexibility, respectively; white blocks represent no data due to absence of a particular CSC in that species. Note: The calculated hierarchical clustering of the *Drosophila* species based on inter-clustal spacing is in near but not complete agreement with their predicted evolutionary distance from *D. melanogaster*. (TIFF 1668 kb) Additional file 4: Table S1. cis-regulatory activity of the pdmlocus enhancers. (DOC 259 kb) Additional file 5: Table S2. Shared SOG enhancer conserved DNA elements (5-->3’). (DOC 192 kb) Additional file 6: Figure S4. Clustering of larval neural pdm locus enhancers. Independent enhancer-reporter transgene analysis of nub-8 through nub-13 conserved sequence clusters (CSCs) reveal enhancer function in the third instar larvae during larval CNS development. Shown are ventral views of third instar larval brains and ventral nerve cord (anterior up) where enhancer activity is detected via membrane tagged mCD8-GFP expression (green). **A)** nub-8 regulates expression in a subset of anterior and posterior ventral nerve cord (VNC) neurons **B)** nub-9 drives expression mostly in a subset of lateral VNC neurons. **C)** nub-10 regulates expression in a subset of lateral VNC neurons. **D)** nub-11 directs expression in a subset of anterolateral VNC and central brain neurons. **E)** nub-12 drives expression in a subset of posterolateral VNC neurons. **F)** nub-13 regulates expression in a subset of posterolateral VNC neurons. Note: The expression patterns of additional *pdm* locus enhancers are shown on the *cis*Patterns website. (TIFF 9357 kb) Additional file 7: Figure S5. Genomic location of dm locus NB enhancers. A genomic schematic of the adjacent *pdm* genes on the 2nd chromosome. The orange boxes represent the nub-46, nub-49a, pdm2-34, and pdm2-37a enhancers that direct NB expression. (TIFF 158 kb)

## Abbreviations

TF: Transcription Factor
NB: Neuroblast
*hb*: *hunchback*
*Kr*: *Krüppel*
*pdm*: *nubbin and pdm-2*
*cas*: *castor*
CSC: conserved sequence cluster
SOG: subesophageal ganglion
*nub*: *nubbin*
Medfly: Mediterranean fruit fly
Housefly: *Musca domestica*
CSBs: Conserved sequence blocks
VNC: Ventral nerve cord
(t): thoracic segment
(a): abdominal segment
CCAP: Cardioacceleratory peptide
mNSCs: median neurosecretory cells

## References

[1] Kohwi M, Doe CQ (2013). Temporal fate specification and neural progenitor competence during development. Nat Rev Neurosci.

[2] Urbach R, Technau GM (2004). Neuroblast formation and patterning during early brain development in Drosophila. Bioessays.

[3] Kambadur R, Koizumi K, Stivers C, Nagle J, Poole SJ, Odenwald WF (1998). Regulation of POU genes by castor and hunchback establishes layered compartments in the Drosophila CNS. Genes Dev.

[4] Hirono K, Margolis JS, Posakony JW, Doe CQ (2012). Identification of hunchback cis-regulatory DNA conferring temporal expression in neuroblasts and neurons. Gene Expr Patterns.

[5] Kuzin A, Kundu M, Ross J, Koizumi K, Brody T, Odenwald WF (2012). The cis-regulatory dynamics of the Drosophila CNS determinant castor are controlled by multiple sub-pattern enhancers. Gene Expr Patterns.

[6] Davidson EH, Erwin DH (2006). Gene regulatory networks and the evolution of animal body plans. Science.

[7] Bergman CM, Pfeiffer BD, Rincon-Limas DE, Hoskins RA, Gnirke A, Mungall CJ, et al*.* Assessing the impact of comparative genomic sequence data on the functional annotation of the Drosophila genome. Genome Biol*.* 2002;3(12):RESEARCH0086. 10.1186/gb-2002-3-12-research0086 PMC151188 12537575

[8] Brody T, Yavatkar AS, Kuzin A, Kundu M, Tyson LJ, Ross J, Lin TY, Lee CH, Awasaki T, Lee T (2012). Use of a Drosophila genome-wide conserved sequence database to identify functionally related cis-regulatory enhancers. Dev Dyn.

[9] Berman BP, Pfeiffer BD, Laverty TR, Salzberg SL, Rubin GM, Eisen MB, Celniker SE (2004). Computational identification of developmental enhancers: conservation and function of transcription factor binding-site clusters in Drosophila melanogaster and Drosophila pseudoobscura. Genome Biol.

[10] Billin AN, Cockerill KA, Poole SJ (1991). Isolation of a family of Drosophila POU domain genes expressed in early development. Mech Dev.

[11] Lloyd A, Sakonju S (1991). Characterization of two Drosophila POU domain genes, related to oct-1 and oct-2, and the regulation of their expression patterns. Mech Dev.

[12] Dick T, Yang XH, Yeo SL, Chia W (1991). Two closely linked Drosophila POU domain genes are expressed in neuroblasts and sensory elements. Proc Natl Acad Sci U S A.

[13] Treisman J, Desplan C (1989). The products of the Drosophila gap genes hunchback and Kruppel bind to the hunchback promoters. Nature.

[14] Stanojevic D, Hoey T, Levine M (1989). Sequence-specific DNA-binding activities of the gap proteins encoded by hunchback and Kruppel in Drosophila. Nature.

[15] Yeo SL, Lloyd A, Kozak K, Dinh A, Dick T, Yang X, Sakonju S, Chia W (1995). On the functional overlap between two Drosophila POU homeo domain genes and the cell fate specification of a CNS neural precursor. Genes Dev.

[16] Beverley SM, Wilson AC (1984). Molecular evolution in Drosophila and the higher Diptera II. A time scale for fly evolution. J Mol Evol.

[17] Rohdendorf BB, Hocking B, Oldroyd H, Ball GE (1974). The historical development of diptera.

[18] Altschul SF, Gish W, Miller W, Myers EW, Lipman DJ (1990). Basic local alignment search tool. J Mol Biol.

[19] Kuzin A, Kundu M, Ekatomatis A, Brody T, Odenwald WF (2009). Conserved sequence block clustering and flanking inter-cluster flexibility delineate enhancers that regulate nerfin-1 expression during Drosophila CNS development. Gene Expr Patterns.

[20] Russo CA, Takezaki N, Nei M (1995). Molecular phylogeny and divergence times of drosophilid species. Mol Biol Evol.

[21] Spicer GS (1991). Molecular evolution and phylogeny of the Drosophila virilis species group as inferred by two-dimensional electrophoresis. J Mol Evol.

[22] Graveley BR, Brooks AN, Carlson JW, Duff MO, Landolin JM, Yang L, Artieri CG, van Baren MJ, Boley N, Booth BW (2011). The developmental transcriptome of Drosophila melanogaster. Nature.

[23] Lundell MJ, Hirsh J (1998). eagle is required for the specification of serotonin neurons and other neuroblast 7–3 progeny in the Drosophila CNS. Development.

[24] Tran KD, Doe CQ (2008). Pdm and Castor close successive temporal identity windows in the NB3-1 lineage. Development.

[25] Ng M, Diaz-Benjumea FJ, Cohen SM (1995). Nubbin encodes a POU-domain protein required for proximal-distal patterning in the Drosophila wing. Development.

[26] Groth AC, Calos MP (2004). Phage integrases: biology and applications. J Mol Biol.

[27] Bhat KM, Apsel N (2004). Upregulation of Mitimere and Nubbin acts through cyclin E to confer self-renewing asymmetric division potential to neural precursor cells. Development.

[28] Bhat KM, Poole SJ, Schedl P (1995). The miti-mere and pdm1 genes collaborate during specification of the RP2/sib lineage in Drosophila neurogenesis. Mol Cell Biol.

[29] Pfeiffer BD, Ngo TT, Hibbard KL, Murphy C, Jenett A, Truman JW, Rubin GM (2010). Refinement of tools for targeted gene expression in Drosophila. Genetics.

[30] Duffy JB (2002). GAL4 system in Drosophila: a fly geneticist's Swiss army knife. Genesis.

[31] Santos JG, Vomel M, Struck R, Homberg U, Nassel DR, Wegener C (2007). Neuroarchitecture of peptidergic systems in the larval ventral ganglion of Drosophila melanogaster. PLoS One.

[32] Perez E (2007). Genetic analysis of cell specification in the Drosophila serotonergic lineage.

[33] Ng M, Diaz-Benjumea FJ, Vincent JP, Wu J, Cohen SM (1996). Specification of the wing by localized expression of wingless protein. Nature.

[34] Lecuit T, Cohen SM (1997). Proximal-distal axis formation in the Drosophila leg. Nature.

[35] French V, Daniels G (1994). Pattern formation. The beginning and the end of insect limbs. Curr Biol.

[36] Held LI (2002). Imaginal discs : the genetic and cellular logic of pattern formation.

[37] Zhu S, Wildonger J, Barshow S, Younger S, Huang Y, Lee T (2012). The bHLH repressor Deadpan regulates the self-renewal and specification of Drosophila larval neural stem cells independently of Notch. PLoS One.

[38] Nassel DR, Kubrak OI, Liu Y, Luo J, Lushchak OV (2013). Factors that regulate insulin producing cells and their output in. Front Physiol.

[39] Farris SM (2008). Tritocerebral tract input to the insect mushroom bodies. Arthropod Struct Dev.

[40] Wang Z, Singhvi A, Kong P, Scott K (2004). Taste representations in the Drosophila brain. Cell.

[41] Seelig JD, Jayaraman V (2013). Feature detection and orientation tuning in the Drosophila central complex. Nature.

[42] Hanesch U, Fischbach K, Heisenberg M (1989). Neuronal architecture of the central complex in Drosophila melanogaster. Cell Tissue Res.

[43] Jenett A, Rubin GM, Ngo TT, Shepherd D, Murphy C, Dionne H, Pfeiffer BD, Cavallaro A, Hall D, Jeter J (2012). A GAL4-driver line resource for Drosophila neurobiology. Cell Rep.

[44] Manning L, Heckscher ES, Purice MD, Roberts J, Bennett AL, Kroll JR, Pollard JL, Strader ME, Lupton JR, Dyukareva AV (2012). A resource for manipulating gene expression and analyzing cis-regulatory modules in the Drosophila CNS. Cell Rep.

[45] Jory A, Estella C, Giorgianni MW, Slattery M, Laverty TR, Rubin GM, Mann RS (2012). A survey of 6,300 genomic fragments for cis-regulatory activity in the imaginal discs of Drosophila melanogaster. Cell Rep.

[46] Pfeiffer BD, Jenett A, Hammonds AS, Ngo TT, Misra S, Murphy C, Scully A, Carlson JW, Wan KH, Laverty TR (2008). Tools for neuroanatomy and neurogenetics in Drosophila. Proc Natl Acad Sci U S A.

[47] Kvon EZ, Kazmar T, Stampfel G, Yanez-Cuna JO, Pagani M, Schernhuber K, Dickson BJ, Stark A (2014). Genome-scale functional characterization of Drosophila developmental enhancers in vivo. Nature.

[48] Datta RR, Small S (2011). Gene regulation: piecing together the puzzle of enhancer evolution. Curr Biol.

[49] Wittkopp PJ (2006). Evolution of cis-regulatory sequence and function in Diptera. Heredity (Edinb).

[50] Naval-Sanchez M, Potier D, Haagen L, Sanchez M, Munck S, Van de Sande B, Casares F, Christiaens V, Aerts S (2013). Comparative motif discovery combined with comparative transcriptomics yields accurate targetome and enhancer predictions. Genome Res.

[51] Rouault H, Santolini M, Schweisguth F, Hakim V (2014). Imogene: identification of motifs and cis-regulatory modules underlying gene co-regulation. Nucleic Acids Res.

[52] Potier D, Seyres D, Guichard C, Iche-Torres M, Aerts S, Herrmann C, Perrin L (2014). Identification of cis-regulatory modules encoding temporal dynamics during development. BMC Genomics.

[53] Barolo S, Castro B, Posakony JW (2004). New Drosophila transgenic reporters: insulated P-element vectors expressing fast-maturing RFP. Biotechniques.

[54] Markstein M, Pitsouli C, Villalta C, Celniker SE, Perrimon N (2008). Exploiting position effects and the gypsy retrovirus insulator to engineer precisely expressed transgenes. Nat Genet.

[55] Ashburner M (1989). Drosophila.

[56] Tomancak P, Beaton A, Weiszmann R, Kwan E, Shu S, Lewis SE, et al. Systematic determination of patterns of gene expression during Drosophila embryogenesis. Genome Biol. 2002;3(12):RESEARCH0088. 10.1186/gb-2002-3-12-research0088 PMC151190 12537577

[57] Campos-Ortega JA, Hartenstein V (1985). The embryonic development of Drosophila melanogaster.

[58] Morey M, Yee SK, Herman T, Nern A, Blanco E, Zipursky SL (2008). Coordinate control of synaptic-layer specificity and rhodopsins in photoreceptor neurons. Nature.

[59] Welling L, Thomson L (2008). PHP and MySQL Web development.

