# Supplementary figures and images for: cis-regulatory analysis of the Drosophila pdm locus reveals a diversity of neural enhancers
Source: BMC Genomics. 2015 Sep 16;16(1):700. doi: 10.1186/s12864-015-1897-2 (PMC4574355; doi:10.1186/s12864-015-1897-2)

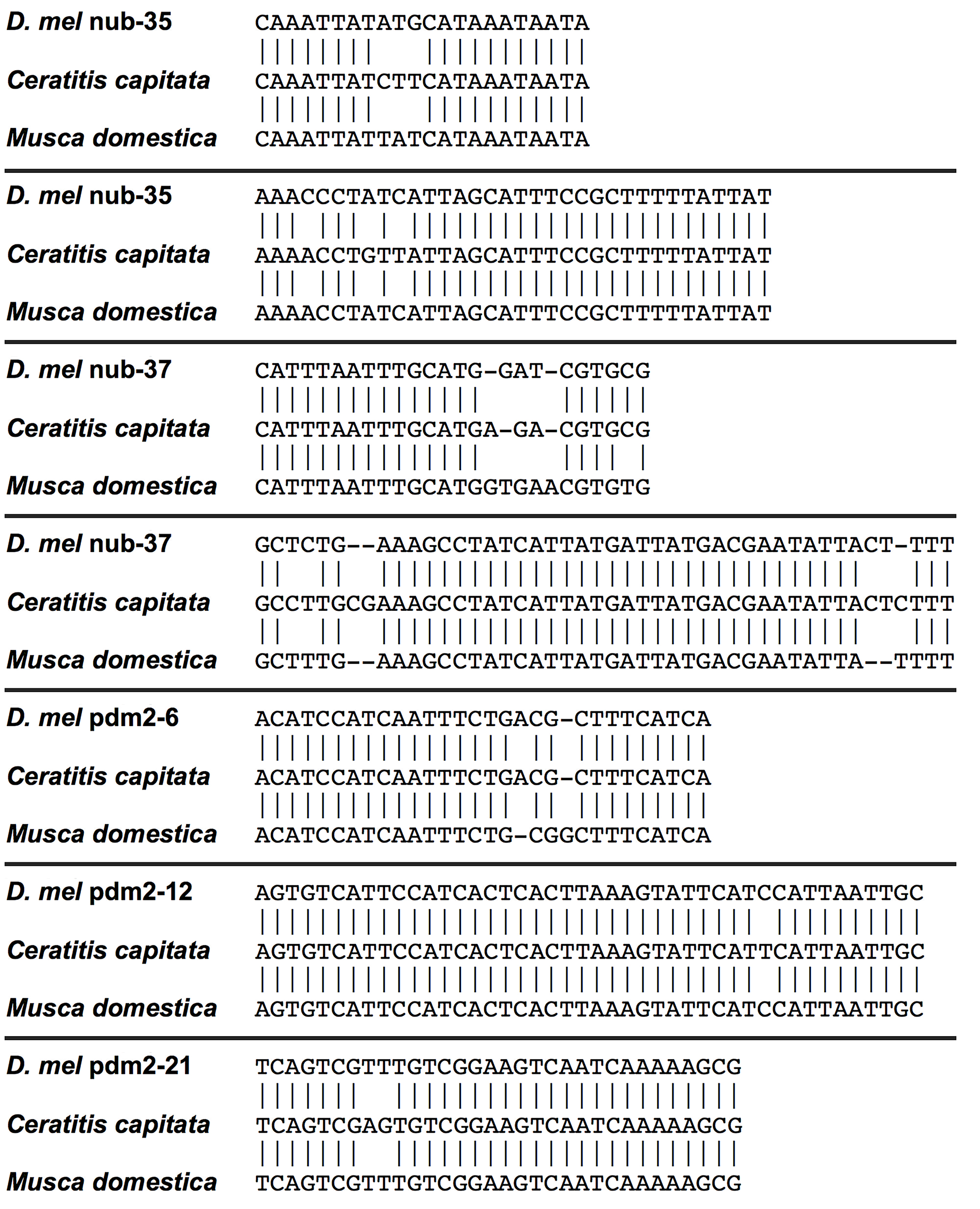

Supplement: Additional file 1: Figure S1. — Three-way alignment of ultraconserved sequences in conserved sequence clusters identified in Drosophila, housefly, and medfly. Shown are conserved sequences shared in Drosophila conserved sequence clusters (nub-35, nub-37, pdm2-6, pdm2-12, and pdm2-21) and detected in the housefly (Musca domestica) and medfly (Ceratitis capitata). Vertical lines indicate agreement among all three Diptera. (TIFF 6544 kb) [file 12864_2015_1897_MOESM1_ESM.tif]

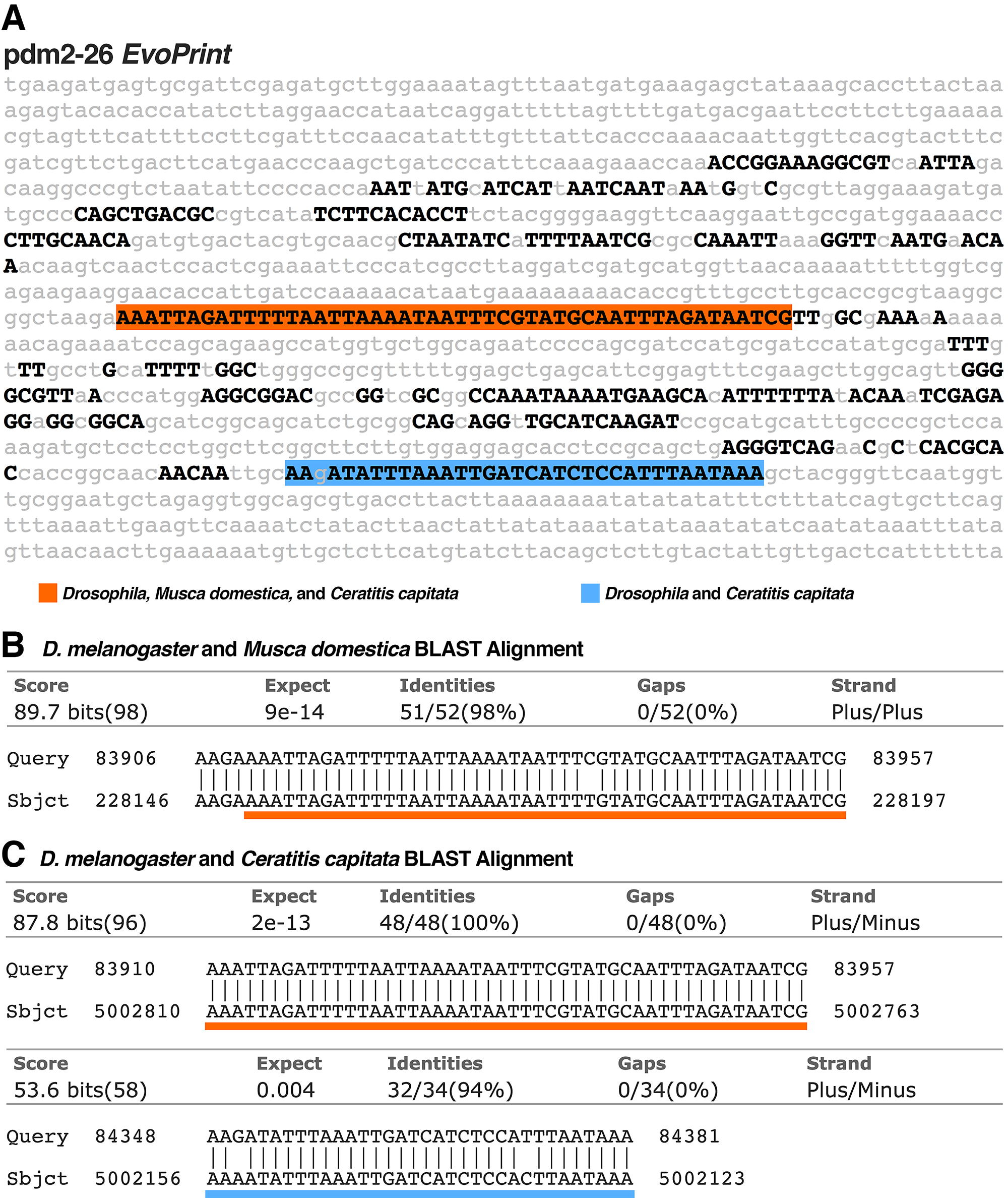

Supplement: Additional file 2: Figure S2. — The pdm2-26 enhancer contains ultraconserved sequences detected in multiple Diptera. A) Shown is a 12-drosophilid EvoPrint of the pdm2-26 enhancer sequence together with colored highlights that indicate conserved D. melanogaster sequences shared with both the housefly and medfly (Musca domestica and Ceratitis capitata, respectively; orange) or with the medfly only (blue). Black capital letters represent D. melanogaster bases conserved in D. simulans, D. sechellia, D. yakuba, D. erecta, D. ananassae, D. persimilis, D. pseudoobscura, D. virilis, and D. mojavensis. B) and C) Raw BLAST results of the pdm2-26 conserved elements aligned to the housefly (Musca domesticus) and medfly (Ceratitis capitata) genomes, respectively. The colored underlines correspond to the colored highlighted conserved sequences in panel A). (TIFF 7785 kb) [file 12864_2015_1897_MOESM2_ESM.tif]

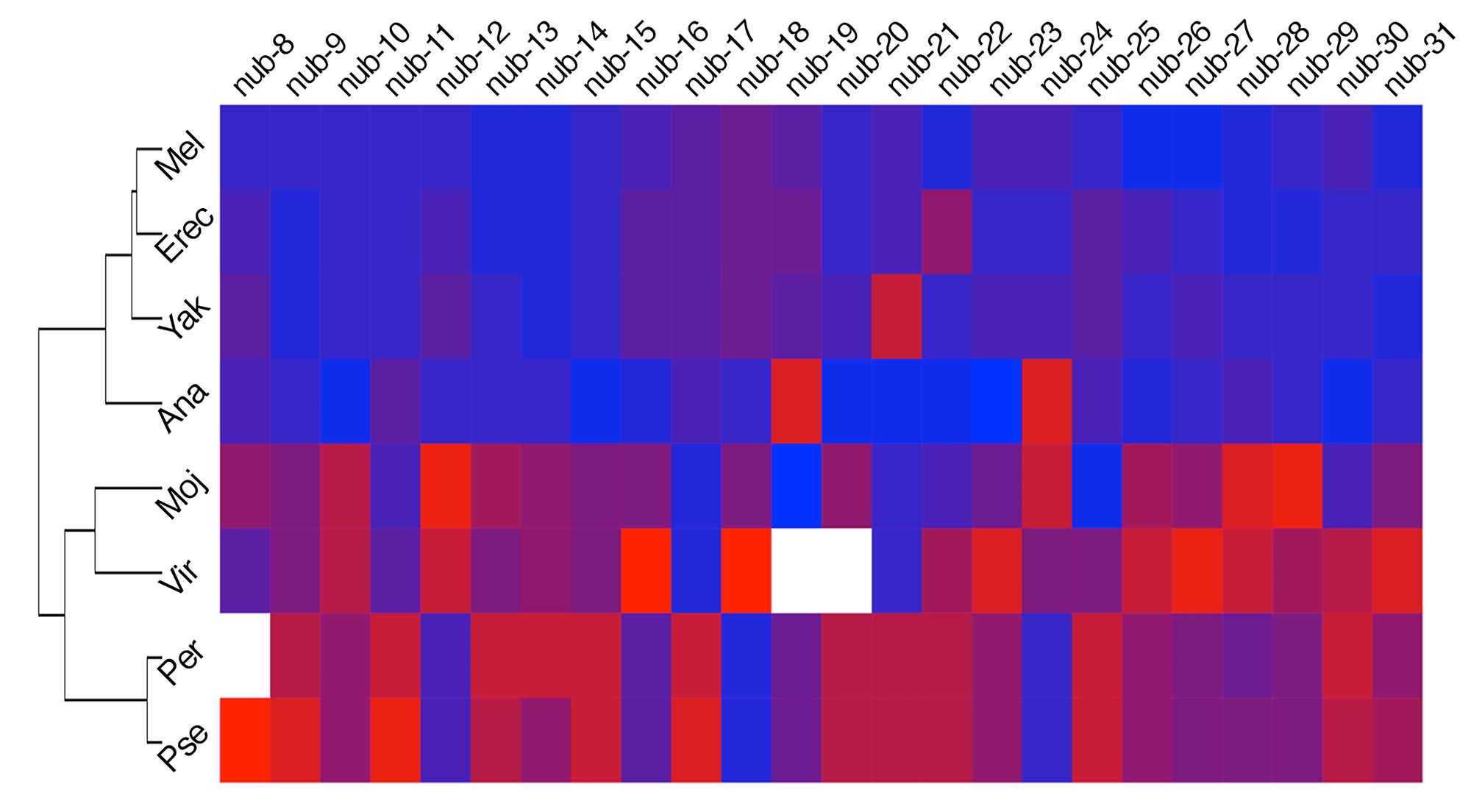

Supplement: Additional file 3: Figure S3. — Resolution of conserved sequence clusters is enhanced by evolutionary flexibility in their flanking less-conserved DNA. Shown is a dendrogram tree that illustrates the similarity among Drosophila species based on the inter-clustal spacing of their conserved sequence clusters (CSCs). This also includes 24 consecutive CSCs across 8 Drosophila species (D. melanogaster, Mel; D. erecta, Erec; D. yakuba, Yak; D. ananassae, Ana; D. mojavensis, Moj; D. virilis, Vir; D. persimilis, Per; and D. pseudoobscura, Pse). The heat map represents a visualization of inter-clustal spacing flexibility, where blue and red blocks highlight lower and higher inter-clustal spacing flexibility, respectively; white blocks represent no data due to absence of a particular CSC in that species. Note: The calculated hierarchical clustering of the Drosophila species based on inter-clustal spacing is in near but not complete agreement with their predicted evolutionary distance from D. melanogaster. (TIFF 1668 kb) [file 12864_2015_1897_MOESM3_ESM.tif]

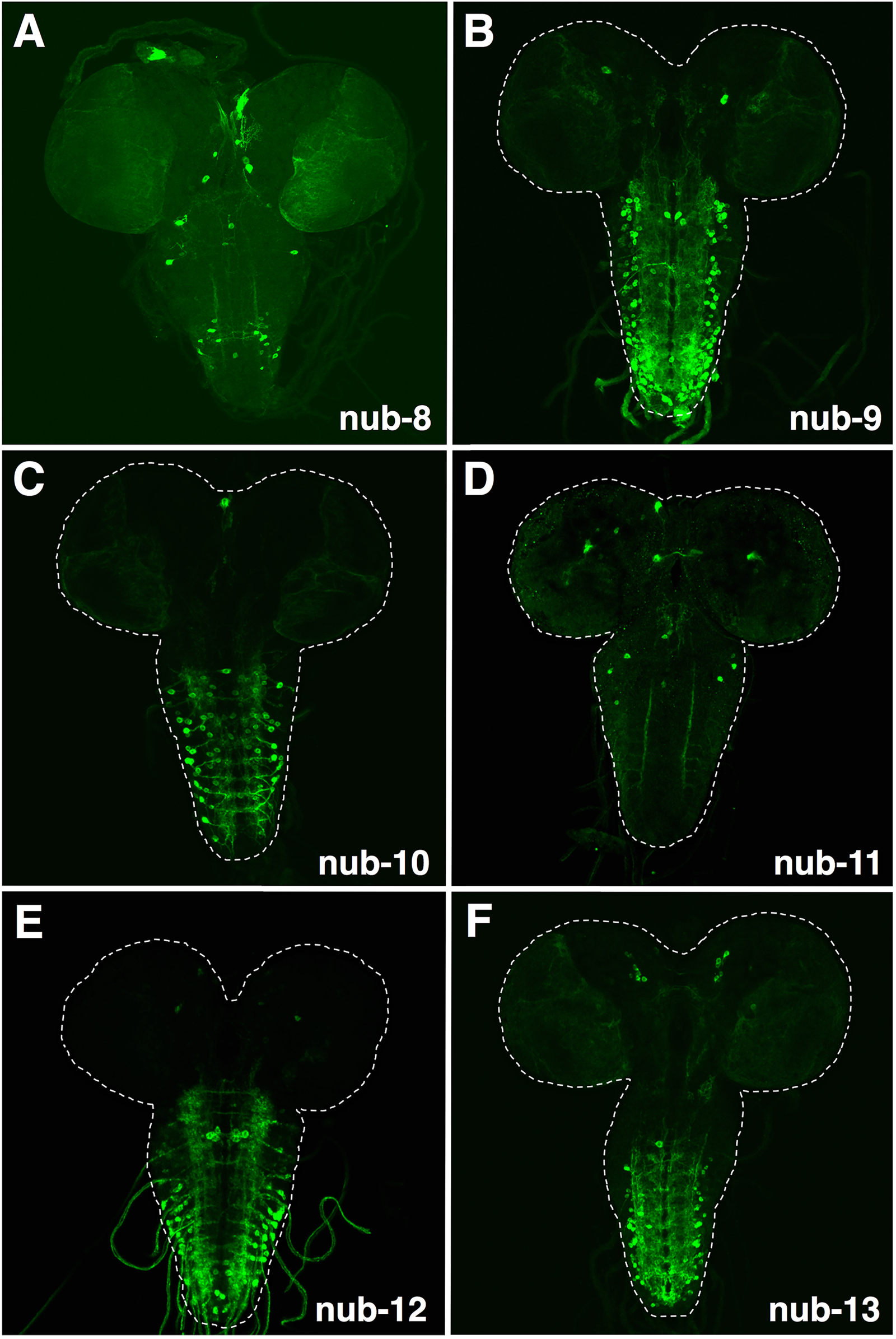

Supplement: Additional file 6: Figure S4. — Clustering of larval neural pdm locus enhancers. Independent enhancer-reporter transgene analysis of nub-8 through nub-13 conserved sequence clusters (CSCs) reveal enhancer function in the third instar larvae during larval CNS development. Shown are ventral views of third instar larval brains and ventral nerve cord (anterior up) where enhancer activity is detected via membrane tagged mCD8-GFP expression (green). A) nub-8 regulates expression in a subset of anterior and posterior ventral nerve cord (VNC) neurons B) nub-9 drives expression mostly in a subset of lateral VNC neurons. C) nub-10 regulates expression in a subset of lateral VNC neurons. D) nub-11 directs expression in a subset of anterolateral VNC and central brain neurons. E) nub-12 drives expression in a subset of posterolateral VNC neurons. F) nub-13 regulates expression in a subset of posterolateral VNC neurons. Note: The expression patterns of additional pdm locus enhancers are shown on the cisPatterns website. (TIFF 9357 kb) [file 12864_2015_1897_MOESM6_ESM.tif]

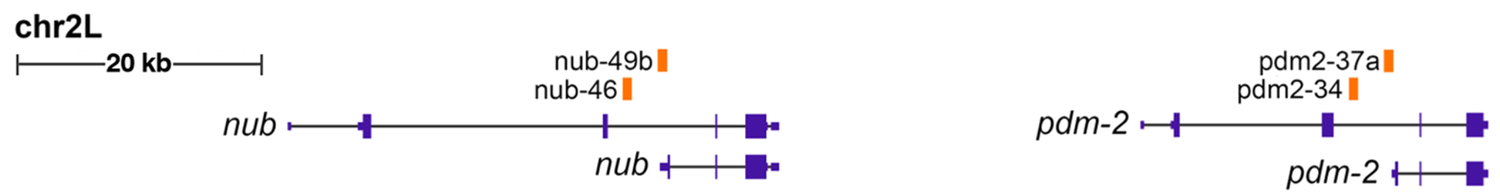

Supplement: Additional file 7: Figure S5. — Genomic location of dm locus NB enhancers. A genomic schematic of the adjacent pdm genes on the 2nd chromosome. The orange boxes represent the nub-46, nub-49a, pdm2-34, and pdm2-37a enhancers that direct NB expression. (TIFF 158 kb) [file 12864_2015_1897_MOESM7_ESM.tif]
