# Supplementary material for: cis-regulatory analysis of the Drosophila pdm locus reveals a diversity of neural enhancers
Source: BMC Genomics. 2015 Sep 16;16(1):700. doi: 10.1186/s12864-015-1897-2 (PMC4574355; doi:10.1186/s12864-015-1897-2)
Supplement: Additional file 4: Table S1. — cis-regulatory activity of the pdmlocus enhancers. (DOC 259 kb) [file 12864_2015_1897_MOESM4_ESM.doc]

| **Table S1: *cis*-regulatory activity of the *pdm* locus enhancers** | | | | | |
| --- | --- | --- | --- | --- | --- |
| **CSC** | **Genomic Location** | **Size (bp)** | **Regulatory Activity During Development** | | |
|  |  |  | **Embryonic** | **Larval** | **Adult** |
| **nub-8** | chr2L:12,565,558-12,566,561 | 1004 | No expression | Anteromedial, Posterolateral, Posteromedial VNC neurons | Ventrolateral Protocerebrum, Ventrolmedial Protocerebrum, Superior Dorsofrontal Protocerebrum |
| **nub-9** | chr2L:12,566,359-12,567,353 | 995 | Stage 15, VNC cells | Anterolateral and Posterolateral VNC neurons | Mushroom Body, Median Neurosecretory Cells, Subesophageal Ganglion, Lateral Horn, Ventrolateral Protocerebrum |
| **nub-10** | chr2L:12,567,302-12,568,686 | 1385 | No expression | Anterolateral and Posterolateral VNC neurons | No expression |
| **nub-11** | chr2L:12,568,904-12,569,530 | 627 | Stage 13, Anterior midgut rudiment | Medial central brain neurons | Antennal Lobe, Subesophageal Ganglion, Medulla, Median Neurosecretory Cells, Ventrolateral Protocerebrum |
| **nub-12** | chr2L:12,569,636-12,570,369 | 734 | Stage 13, Procephalon cells, PNS cells, VNC cells | Anteromedial and Posterolateral VNC neurons | Median Neurosecretory Cells, Subesophageal Ganglion, Ventrolateral Protocerebrum, Ellipsoid Body, Lateral Triangle, Cell Body |
| **nub-13** | chr2L:12,570,645-12,571,697 | 1053 | No expression | Posterolateral VNC neurons | Median Neurosecretory Cells, Subesophageal Ganglion, Ventrolateral Protocerebrum, Ellipsoid Body, Lateral Triangle |
| **nub-14** | chr2L:12,571,609-12,572,607 | 999 | No expression | No expression | No expression |
| **nub-15** | chr2L:12,572,590-12,573,775 | 1186 | No expression | Lateral central brain neurons, and Posteromedial and Anteromedial VNC neurons | Median Neurosecretory Cells, Ellipsoid Body, Lateral Triangle, Cell Body |
| **nub-16** | chr2L:12,573,773-12,574,672 | 900 | Stage 15, Maxillary bud, Salivary gland | No expression | Median Neurosecretory Cells, Ventrolateral Protocerebrum, Ellipsoid Body, Lateral Triangle, Medulla, Lobula Plate, Lobula Subesophageal Ganglion, Optic Tubercle, Dorsolateral Protocerebrum, Cell Body |
| **nub-17** | chr2L:12,574,720-12,575,730 | 1011 | No expression | Lateral and Medial central brain neurons, Anteromedial, Posterolateral, Posteromedial VNC neurons | No expression |
| **nub-18** | chr2L:12,575,979-12,576,824 | 846 | No expression | No expression | No expression |
| **nub-19** | chr2L:12,576,805-12,578,458 | 1654 | No expression | Leg imaginal disc | No expression |
| **nub-20** | chr2L:12,578,523-12,579,501 | 979 | Stage 13, Maxillary bud, Salivary gland, Procephalon cells, VNC cells | Lateral central brain neurons | No expression |
| **nub-21** | chr2L:12,579,689-12,580,536 | 848 | No expression | No expression | No expression |
| **nub-22-23** | chr2L:12,580,537-12,583,186 | 2650 | No expression | No expression | No expression |
| **nub-24** | chr2L:12,582,987-12,584,589 | 1603 | No expression | No expression | No expression |
| **nub-25** | chr2L:12,584,575-12,585,833 | 1259 | No expression | Medial central brain neurons | No expression |
| **nub-26-27** | chr2L:12,585,731-12,587,849 | 2119 | No expression | No expression | No expression |
| **nub-28** | chr2L:12,588,809-12,589,959 | 1151 | Stage 15, Midgut, Salivary gland | No expression | No expression |
| **nub-29** | chr2L:12,590,702-12,592,039 | 1338 | Stage 14, Amnioserosa | No expression | Trachea |
| **nub-30** | chr2L:12,591,852-12,593,034 | 1183 | Stage 14, Clypeolabrum, Esophagus, Salivary gland | No expression | No expression |
| **nub-31** | chr2L:12,592,923-12,593,999 | 1077 | Stage 12, Clypeolabrum, Amnioserosa | Leg imaginal disc | Antennal Lobe, Antennal Mechanosensory and Motor Center, Median Neurosecretory Cells, Subesophageal Ganglion Ventrolateral Protocerebrum, Dorsolateral Protocerebrum, Optic Tubercle |
| **nub-32a** | chr2L:12,593,989-12,594,734 | 746 | Stage 13, Salivary gland | No expression | Median Neurosecretory Cells, Dorsolateral Protocerebrum, Subesophageal Ganglion, Ventrolateral Protocerebrum |
| **nub-32b** | chr2L:12,594,756-12,595,867 | 1112 | Stage 15, Esophagus, Maxillary bud | Leg imaginal disc | No expression |
| **nub-32c** | chr2L:12,595,682-12,596,920 | 1239 | Stage 14, Salivary gland, Epidermis | Posterolateral VNC glial | Subesophageal Ganglion (non-neuronal) |
| **nub-33** | chr2L:12,597,237-12,598,060 | 824 | Stage 15, Cardiac cells, Esophagus | No expression | No expression |
| **nub-34** | chr2L:12,598,239-12,598,947 | 709 | No expression | No expression | No expression |
| **nub-35** | chr2L:12,598,981-12,600,252 | 1272 | No expression | No expression | No expression |
| **nub-36** | chr2L:12,600,281-12,601,211 | 931 | No expression | Leg imaginal disc | No expression |
| **nub-37-40a** | chr2L:12,601,864-12,605,036 | 3173 | No expression | No expression | No expression |
| **nub-40b** | chr2L:12,605,017-12,605,705 | 688 | No expression | No expression | Ubiquitous central brain |
| **nub-41** | chr2L:12,605,933-12,607,774 | 1845 | Stage 10, Procephalic neuroblasts | Haltere and Wing imaginal discs | No expression |
| **nub-42** | chr2L:12,607,948-12,609,464 | 1517 | Stage 13, Esophagus, VNC cells | Optic Lobe and Anterolateral VNC neurons | No expression |
| **nub-43** | chr2L:12,609,443-12,610,996 | 1554 | No expression | No expression | No expression |
| **nub-44** | chr2L:12,610,975-12,612,649 | 1675 | Stage 9, Procephalic neuroblasts | No expression | No expression |
| **nub-45a** | chr2L:12,612,630-12,613,572 | 943 | No expression | Posteromedial VNC neurons | No expression |
| **nub-45b** | chr2L:12,613,552-12,614,521 | 970 | No expression | No expression | Dorsolateral Protocerebrum, Ventrolateral Protocerebrum |
| **nub-46** | chr2L:12,614,496-12,615,368 | 873 | Stage 11, Procephalic neuroblasts, VNC cells and neuroblasts | Medial and Lateral central brain neuroblasts GMCs, and neurons; Anterolateral and Anteromedial VNC neuroblasts | Optic Glomerulus |
| **nub-47-48** | chr2L:12,615,351-12,617,354 | 2004 | Stage 7, Cellular blastoderm | No expression | No expression |
| **nub-49a** | chr2L:12,617,335-12,618,354 | 1020 | No expression | No expression | No expression |
| **nub-49b** | chr2L:12,618,337-12,618,819 | 483 | Stage 12, Procephalon, VNC cells | Lateral central brain neuroblasts, GMCs, neurons; Anteromedial, Posterolateral, and Posteromedial VNC neurons | Medulla, Lobula Plate, Subesophageal Ganglion, Antennal Lobe, Antennal Mechanosensory and Motor Center, Mushroom Body, Median Neurosecretory Cells, Dorsolateral Protocerebrum, Inferior Dorsofrontal Protocerebrum |
| **nub-50** | chr2L:12,618,801-12,619,614 | 814 | No expression | No expression | No expression |
| **nub-51** | chr2L:12,619,677-12,620,397 | 721 | No expression | No expression | No expression |
| **nub-52** | chr2L:12,620,577-12,621,240 | 664 | No expression | No expression | Trachea |
| **nub-53** | chr2L:12,621,241-12,622,288 | 1048 | Stage 10, VNC cells | Optic Lobe, Lateral, and Medial central brain neurons; Anterolateral and Anteromedial VNC neurons | Medulla, Lobula Plate, Subesophageal Ganglion, Antennal Lobe, Antennal Mechanosensory and Motor Center, Median Neurosecretory Cells, Dorsolateral Protocerebrum, Inferior Dorsofrontal Protocerebrum |
| **nub-54** | chr2L:12,622,311-12,624,179 | 1869 | Stage 14, Cardiac cells | Medial central brain neurons; Anterolateral and Posterolateral VNC neurons; Posterolateral and Posteromedial VNC glial | Noduli, Median Neurosecretory Cells |
| **nub-55-56** | chr2L:12,624,192-12,627,085 | 2894 | No expression | No expression | No expression |
| **nub-57** | chr2L:12,627,096-12,628,319 | 1224 | No expression | No expression | No expression |
| **nub-58a** | chr2L:12,628,297-12,628,765 | 469 | Stage 13, Cardiac cells | No expression | Medulla, Lobula Plate, Subesophageal Ganglion, Antennal Lobe, Antennal Mechanosensory and Motor Center, Median Neurosecretory Cells, Dorsolateral Protocerebrum |
| **nub-58b** | chr2L:12,628,766-12,629,210 | 445 | Stage 13, Amnioserosa | Posteromedial VNC neurons and glial | No expression |
| **pdm2-1** | chr2L:12,629,177-12,630,200 | 1024 | No expression | No expression | No expression |
| **pdm2-2a** | chr2L:12,630,430-12,631,327 | 898 | No expression | No expression | No expression |
| **pdm2-2b** | chr2L:12,631,308-12,632,017 | 710 | No expression | Posteromedial VNC neurons | No expression |
| **pdm2-3** | chr2L:12,631,998-12,632,582 | 585 | No expression | No expression | No expression |
| **pdm2-4** | chr2L:12,632,563-12,633,837 | 1275 | No expression | No expression | No expression |
| **pdm2-5a** | chr2L:12,633,852-12,634,629 | 778 | Stage 13, Salivary gland | Anteromedial VNC neurons | No expression |
| **pdm2-5b** | chr2L:12,634,625-12,635,502 | 878 | Stage 13, Amnioserosa | No expression | No expression |
| **pdm2-6** | chr2L:12,635,483-12,636,578 | 1096 | No expression | Medial and Lateral central brain neurons | Median Neurosecretory Cells, Caudal Ventrolateral Protocerebrum, Super-peduncular protocerebrum |
| **pdm2-7a** | chr2L:12,636,560-12,637,029 | 470 | No expression | No expression | No expression |
| **pdm2-7b** | chr2L:12,637,010-12,638,221 | 1212 | Stage 10, Procephalic neuroblasts | Medial central brain and Posterolateral VNC neurons | Antennal Lobe |
| **pdm2-8a** | chr2L:12,639,078-12,639,970 | 893 | No expression | No expression | No expression |
| **pdm2-8b** | chr2L:12,639,952-12,640,656 | 705 | No expression | No expression | Median Neurosecretory Cells, Optic Glomerulus |
| **pdm2-8c** | chr2L:12,640,639-12,642,047 | 1409 | Stage 13, Amnioserosa | No expression | No expression |
| **pdm2-9** | chr2L:12,642,243-12,643,334 | 1092 | Stage 13, Cardiac cells, PNS cells | Midline, Anteromedial, and Posteromedial VNC neurons | No expression |
| **pdm2-10** | chr2L:12,643,774-12,644,395 | 622 | No expression | No expression | Trachea |
| **pdm2-11** | chr2L:12,644,376-12,645,619 | 1244 | No expression | Medial and Lateral central brain neurons; Anterolateral, Anteromedial, Posterolateral, Posteromedial VNC neurons | No expression |
| **pdm2-12a** | chr2L:12,645,600-12,646,414 | 815 | No expression | No expression | No expression |
| **pdm2-12b** | chr2L:12,646,395-12,647,024 | 630 | No expression | No expression | No expression |
| **pdm2-13** | chr2L:12,647,554-12,648,214 | 661 | No expression | Medial and Lateral central brain neurons; Anteromedial, Posterolateral, and Posteromedial VNC neurons | Subesophageal Ganglion, Dorsolateral Protocerebrum, Median Neurosecretory Cells, Lobula Plate, Medulla, Lateral Horn |
| **pdm2-14** | chr2L:12,648,298-12,649,560 | 1263 | No expression | No expression | No expression |
| **pdm2-15** | chr2L:12,649,832-12,650,737 | 906 | Stage 13, Procephalon and VNC cells | Anteromedial and Posterolateral VNC neurons | Laternal Horn |
| **pdm2-16a-16b** | chr2L:12,650,843-12,652,219 | 1377 | No expression | No expression | No expression |
| **pdm2-17** | chr2L:12,652,317-12,653,839 | 1523 | No expression | Medial and Lateral central brain neurons; Anterolateral and Posterolateral VNC neurons | Antennal Lobe, Antennal Mechanosensory and Motor Center, Subesophageal Ganglion, Superior Dorsofrontal Protocerebrum, Optic Glomerulus, Lobula Plate, Medulla, Lateral Horn, Median Neurosecretory Cells, Mushroom Body |
| **pdm2-18** | chr2L:12,653,815-12,654,940 | 1126 | No expression | Medial and Lateral central brain neurons; Anterolateral and Posterolateral VNC neurons | Antennal Lobe, Antennal Mechanosensory and Motor Center, Subesophageal Ganglion, Superior Dorsofrontal Protocerebrum, Optic Glomerulus, Lobula Plate, Medulla, Lateral Horn, Median Neurosecretory Cells, Dorsolateral Protocerebrum |
| **pdm2-19** | chr2L:12,655,010-12,656,495 | 1486 | No expression | Medial and Lateral central brain neurons; Anterolateral, Anteromedial, Posterolateral, and Posteromedial VNC neurons | Antennal Lobe, Subesophageal Ganglion, Superior Dorsofrontal Protocerebrum, Optic Glomerulus, Medulla, Lateral Horn, Median Neurosecretory Cells, Dorsolateral Protocerebrum, Optic Tubercle |
| **pdm2-20** | chr2L:12,656,477-12,657,225 | 749 | No expression | No expression | No expression |
| **pdm2-21** | chr2L:12,657,203-12,658,158 | 956 | Stage 13, PNS cells, Primordia | Anterolateral and Posterolateral VNC | Subesophageal Ganglion, Ventrolmedial Protocerebrum |
| **pdm2-22** | chr2L:12,658,617-12,659,768 | 1152 | Stage 14, Hindgut, Midgut | No expression | No expression |
| **pdm2-23a** | chr2L:12,659,932-12,660,487 | 556 | No expression | No expression | Subesophageal Ganglion, Median Neurosecretory Cells, Lateral Horn |
| **pdm2-23b** | chr2L:12,660,463-12,661,068 | 606 | No expression | No expression | No expression |
| **pdm2-24** | chr2L:12,661,260-12,662,398 | 1139 | No expression | No expression | Subesophageal Ganglion |
| **pdm2-25** | chr2L:12,662,639-12,663,772 | 1134 | Stage 13, Procephalic neuroblasts | No expression | No expression |
| **pdm2-26** | chr2L:12,663,746-12,664,966 | 1221 | Stage 11, Procephalon cells | No expression | No expression |
| **pdm2-27** | chr2L:12,664,951-12,665,519 | 569 | No expression | Anteromedial and Posteromedial VNC glial | Laternal Horn, Subesophageal Ganglion, Antennal Mechanosensory and Motor Center, Median Neurosecretory Cells |
| **pdm2-28** | chr2L:12,665,496-12,666,891 | 1396 | Stage 10, Tracheal pits | No expression | Proximal Antennal Protocerebrum |
| **pdm2-29** | chr2L:12,667,218-12,669,234 | 2017 | No expression | No expression | No expression |
| **pdm2-30** | chr2L:12,669,307-12,669,943 | 637 | No expression | No expression | Glia |
| **pdm2-31a** | chr2L:12,669,923-12,671,165 | 1243 | Stage 12, Procephalic neuroblasts | No expression | Ellipsoid Body, Cell Body, Medulla, Ventromedial Protocerebrum |
| **pdm2-31b** | chr2L:12,671,386-12,672,225 | 840 | Stage 13, Maxillary bud | No expression | No expression |
| **pdm2-32** | chr2L:12,672,188-12,673,706 | 1519 | Stage 13, PNS cells | No expression | Antennal Mechanosensory and Motor Center |
| **pdm2-33** | chr2L:12,673,874-12,674,765 | 892 | No expression | No expression | Caudalcentral Protocerebrum |
| **pdm2-34** | chr2L:12,674,952-12,675,905 | 954 | Stage 11, Procephalic neuroblasts, VNC cells and neuroblasts | Medial and Lateral central brain neuroblasts, GMCs, and neurons; Anterolateral and Anteromedial VNC neuroblasts | No expression |
| **pdm2-35** | chr2L:12,676,032-12,676,949 | 918 | No expression | Medial central brain and Posteromedial VNC neurons | No expression |
| **pdm2-36** | chr2L:12,677,124-12,677,690 | 567 | No expression | No expression | Lobula Plate, Lobula, Optic Glomerulus, Medulla, Subesophageal Ganglion, Ellipsoid Body, Cell Body, Lateral Triangle, Median Neurosecretory Cells |
| **pdm2-37a** | chr2L:12,677,997-12,678,642 | 646 | Stage 11, Clypeolabrum, Salivary gland, Subesophageal ganglion | Optic Lobe and Lateral central brain neuroblasts, GMCs, neurons; Anterolateral VNC neuroblasts | Ubitiquous |
| **pdm2-37b** | chr2L:12,678,622-12,678,983 | 362 | No expression | No expression | Median Neurosecretory Cells |
| **pdm2-38** | chr2L:12,678,965-12,680,422 | 1458 | No expression | No expression | No expression |
| **pdm2-39** | chr2L:12,680,398-12,680,876 | 479 | No expression | Posteromedial VNC neurons | Median Neurosecretory Cells, Ellipsoid Body, Lobula Plate, Lobula, Optic Glomerulus, Medulla |
| **pdm2-40a** | chr2L:12,680,862-12,681,540 | 679 | No expression | Posterolateral VNC neurons | No expression |
| **pdm2-40b** | chr2L:12,681,595-12,682,465 | 871 | No expression | Posterolateral VNC neurons | Median Neurosecretory Cells, Subesophageal Ganglion |
| **pdm2-41** | chr2L:12,682,702-12,683,791 | 890 | Stage 12, Procephalon and VNC cells | Lateral central brain neurons | No expression |
| **pdm2-42** | chr2L:12,683,772-12,684,306 | 535 | No expression | Lateral central brain and Posterolateral neurons | Median Neurosecretory Cells, Superior Dorsofrontal Protocerebrum |
| **pdm2-43** | chr2L:12,684,283-12,685,859 | 1577 | No expression | No expression | No expression |
| **pdm2-44** | chr2L:12,685,840-12,686,379 | 540 | No expression | No expression | No expression |
| **pdm2-45** | chr2L:12,686,358-12,687,347 | 990 | Stage 13, Procephalon and VNC cells | No expression | Lobula, Lobula Plate, Antennal Mechanosensory and Motor Center, Lateral Horn, Superior Dorsofrontal Protocerebrum, Dorsomedial Protocerebrum |
| **pdm2-46** | chr2L:12,687,585-12,688,348 | 764 | No expression | No expression | Subesophageal Ganglion, Lateral Horn, Lobula, Lobula Plate |
| **pdm2-47** | chr2L:12,688,473-12,689,213 | 741 | No expression | Medial central brain neurons; Anteromedial, Posterolateral, and Posteromedial VNC neurons | Dorsolateral Protocerebrum, Subesophageal Ganglion, Lobula, Lobula Plate |
| **pdm2-48** | chr2L:12,689,433-12,690,307 | 875 | No expression | Medial central brain and Anterolateral VNC neurons | No expression |
