# Supplementary material for: cis-regulatory analysis of the Drosophila pdm locus reveals a diversity of neural enhancers
Source: BMC Genomics. 2015 Sep 16;16(1):700. doi: 10.1186/s12864-015-1897-2 (PMC4574355; doi:10.1186/s12864-015-1897-2)
Supplement: Additional file 5: Table S2. — Shared SOG enhancer conserved DNA elements (5-->3’). (DOC 192 kb) [file 12864_2015_1897_MOESM5_ESM.doc]

| **Table S2. Shared SOG enhancer conserved DNA elements (5'-->3').** | |
| --- | --- |
| 1 | CACACACA |
| 2 | TGCTGTT |
| 3 | ATCAGCAG |
| 4 | TCAGCAG |
| 5 | GGCAGCA |
| 6 | TCAGCAGC |
| 7 | TCAGCAGCA |
| 8 | ATCAGCAGC |
| 9 | ATCAGCAGCA |
| 10 | GCCAGCAG |
| 11 | GGCAGCAA |
| 12 | CCAGCAG |
| 13 | TCTGAG |
| 14 | ATCTGAG |
| 15 | TCAGCAGCAGCA |
| 16 | ATCAGCAGCAGC |
| 17 | CATCAGCAGCAG |
| 18 | TCAGCAGCAGC |
| 19 | ATCAGCAGCAG |
| 20 | CATCAGCAGCA |
| 21 | CATCAGCAGC |
| 22 | TCAGCAGCAG |
| 23 | GCTGTTGC |
| 24 | CTGTTGC |
| 25 | CTGCTGCA |
| 26 | CAACAGCAACA |
| 27 | AACAGCAACA |
| 28 | AACAGCAAC |
| 29 | CAACAGCAAC |
| 30 | CAACAGCAA |
| 31 | CTGTTGCT |
| 32 | AACAGCAA |
| 33 | TGTGTGTGTGT |
| 34 | GTGTGTGTGT |
| 35 | TGTTATTT |
| 36 | TGTTATT |
| 37 | TGAAATTAA |
| 38 | GAAATTAA |
| 39 | TAAGTT |
| 40 | TCACCATCA |
| 41 | TCACCATC |
| 42 | ACTTAATTT |
| 43 | AACTTAATTT |
| 44 | AAACAGTT |
| 45 | ACATGCAG |
| 46 | ACATGCAGA |
| 47 | TGCTGCTGTT |
| 48 | TGCTGCTGTTG |
| 49 | GCTGCTGTTG |
| 50 | CTGCTGTTG |
| 51 | CTGCTGTT |
| 52 | GCTGCTGTT |
| 53 | ATTGTTGCT |
| 54 | TGCAGCAGCAAC |
| 55 | GCAACAGCAACA |
| 56 | AACAGCAGCAGC |
| 57 | CAACAGCAGCAG |
| 58 | GCAGCAACAATT |
| 59 | TGCAGCAGCAA |
| 60 | AACAGCAGCAG |
| 61 | CAGCAACAATT |
| 62 | GCAGCAACAAT |
| 63 | AGCAACAATT |
| 64 | CAGCAACAAT |
| 65 | GCAACAATT |
| 66 | GCAGCAGCG |
| 67 | GGCAGCAGC |
| 68 | GGCAGCAG |
| 69 | CAGCAGCG |
| 70 | TTGCTGCTGTTG |
| 71 | TTGCTGCTGTT |
| 72 | ACAACAGCAGCA |
| 73 | TCAGCAGCAA |
| 74 | AACAGCAACAAC |
| 75 | CAACAGCAACAA |
| 76 | AGCAACAACAGC |
| 77 | CAGCAACAACAG |
| 78 | AACAGCAACAA |
| 79 | GCAACAACAGC |
| 80 | GCAACAACAG |
| 81 | AGCAACAACAG |
| 82 | TTGCAGC |
| 83 | CATGTCC |
| 84 | GGCATGT |
| 85 | GAATACAA |
| 86 | TTACTTG |
| 87 | GAATACA |
| 88 | AACTAC |
| 89 | AAACTAC |
| 90 | TGTCCATCA |
| 91 | GTCCATCA |
| 92 | TGTCCATC |
| 93 | TGTGAAC |
| 94 | GTGAAC |
| 95 | GTCCATC |
| 96 | TTGTGTTT |
| 97 | AAAATCGA |
| 98 | TCGATTTTATTT |
| 99 | CGATTTTATTT |
| 100 | TCGATTTTATT |
| 101 | GATTTTATTT |
| 102 | CGATTTTATT |
| 103 | TCGATTTTAT |
| 104 | CGATTTTAT |
| 105 | CGATTTTA |
| 106 | TCGATTTTA |
| 107 | CAACAAG |
| 108 | ACGCACAG |
| 109 | AATGTCAA |
| 110 | TGAGCAA |
| 111 | AGCGACAA |
| 112 | GAGCAAT |
| 113 | TGAGCAAT |
| 114 | AAAATTAAG |
| 115 | AAAACGCAT |
| 116 | TAAGTTT |
| 117 | AAAAAACGC |
| 118 | AGGATATC |
| 119 | GCTGAAAG |
| 120 | ACATTTGG |
| 121 | TGAATTTATGAC |
| 122 | GAATTTATGAC |
| 123 | TGAATTTATGA |
| 124 | AATTTATGAC |
| 125 | TGAATTTATG |
| 126 | TTTATGACA |
| 127 | GTTATTAT |
| 128 | ATTTATGAC |
| 129 | GTGCGCC |
| 130 | TGCGCC |
| 131 | CAATAC |
| 132 | CAATACA |
| 133 | TTACAATG |
| 134 | CATTGTA |
| 135 | ACTGCC |
| 136 | TTTTGCATTG |
| 137 | TTGCATTG |
| 138 | TTTGCATTG |
| 139 | TGCAACAT |
| 140 | ACATTTTC |
| 141 | TGTTATTA |
| 142 | TGTATGC |
| 143 | ATTGTAT |
| 144 | AATACAAT |
| 145 | TACTTG |
| 146 | CAACGAT |
| 147 | ACTTGCA |
| 148 | TGCAGTTTT |
| 149 | GCAGTTTT |
| 150 | GCAGTTT |
| 151 | TGCAGTTT |
| 152 | ATTTTCAA |
| 153 | AAAATGAAAT |
| 154 | ATTGGCAG |
| 155 | TTGGCAG |
| 156 | CGGAAATT |
| 157 | CGGAAATTA |
| 158 | TTAATGAA |
| 159 | TTTAATGAA |
| 160 | ATCAATG |
| 161 | GCATAAAC |
| 162 | GCATAAACA |
| 163 | GATTTTAT |
| 164 | TGAACG |
| 165 | GATTTTATT |
| 166 | TATTTTCAA |
| 167 | TGTGTTTT |
| 168 | TTGTGTTTT |
| 169 | AATAAGAA |
| 170 | TAATAAGAA |
| 171 | CTAATGC |
| 172 | TTGCTGCAG |
| 173 | TTTTGCTG |
| 174 | CCATCAGC |
| 175 | CCATCAG |
| 176 | TAAAAGCG |
| 177 | ATTTCTTTTATT |
| 178 | AATGAAATAAAA |
| 179 | AAATGAAATAAA |
| 180 | AAAATGAAATAA |
| 181 | TTTCTTTTATT |
| 182 | ATTTCTTTTAT |
| 183 | ATGAAATAAAA |
| 184 | AATGAAATAAA |
| 185 | AAATGAAATAA |
| 186 | TTCTTTTATT |
| 187 | TTTCTTTTAT |
| 188 | ATTTCTTTTA |
| 189 | TGAAATAAAA |
| 190 | ATGAAATAAA |
| 191 | AATGAAATAA |
| 192 | TCTTTTATT |
| 193 | TTCTTTTAT |
| 194 | TTTCTTTTA |
| 195 | ATTTCTTTT |
| 196 | ATGAAATAA |
| 197 | TCTTTTAT |
| 198 | TCTTTTA |
| 199 | TTCTTTTA |
| 200 | GCATTTTTATT |
| 201 | GCATTTTTATTG |
| 202 | CACTTGTTG |
| 203 | ATAACAATTT |
| 204 | TCACACAC |
| 205 | TCACACACA |
| 206 | AGGAAAATGT |
| 207 | AGGAAAATG |
| 208 | AGAGGAA |
| 209 | AGGAAAAT |
| 210 | ACATGCAAAAC |
| 211 | ATTTTCAAT |
| 212 | ATTGAAAG |
| 213 | ATTGAAAGG |
| 214 | GCACTCG |
| 215 | GATTTTCA |
| 216 | CACGCACA |
| 217 | TGTGTGTGTGTG |
| 218 | GAGGCA |
| 219 | GAGGCAG |
| 220 | GGCAGTC |
| 221 | GGCAGTCA |
| 222 | ATTTATGTG |
| 223 | AAAATAAC |
| 224 | TGTTTTGCT |
| 225 | ATGCCACA |
| 226 | CGATTGA |
| 227 | GTTTTGCT |
| 228 | GGGTCAA |
| 229 | ATGTTAT |
| 230 | CACGCAG |
| 231 | ACACGCAG |
| 232 | AGACACGC |
| 233 | CCGACG |
| 234 | CCGACGA |
| 235 | TTATGTG |
| 236 | TTTATGTG |
| 237 | TTTATGTGC |
| 238 | TATGTGC |
| 239 | TTATGTGC |
| 240 | TGAAATTAATT |
| 241 | CTGCACAAA |
| 242 | CTGCACAA |
| 243 | TGCACAAA |
| 244 | GCACTTA |
| 245 | CAATAAAAAA |
| 246 | CACAATA |
| 247 | TGCAACATTTT |
| 248 | GCAACATTTT |
| 249 | TGCAACATTT |
| 250 | CAACATTTT |
| 251 | GCAACATTT |
| 252 | TTACATTA |
| 253 | ACATTAC |
| 254 | CAACATTT |
